# Integrated surveillance of arboviruses in febrile patients from the Brazilian Amazon reveals complex co-circulation dynamics and hidden viral diversity

**DOI:** 10.1590/0037-8682-0042-2026

**Published:** 2026-07-17

**Authors:** Karolina Jeaneth Solórzano Chavarría, Erika Oliveira Gomes, Bárbara Aparecida Chaves, Vanderson Souza Sampaio, Alexandre Vilhena Silva-Neto, Daniela Brito, Luís Felipe Alho da Silva, Maianne Yasmin Oliveira Dias, Lívia Sacchetto, Victoria Bernardi, Beatriz de Carvalho Marques, Michaela Buenemann, Nikos Vasilakis, Maurício Lacerda Nogueira, Marcus Vinícius Guimarães Lacerda, Maria Paula Gomes Mourão, Djane Clarys Baía-da-Silva

**Affiliations:** 1 Fundação de Medicina Tropical Doutor Heitor Vieira Dourado, Manaus, AM, Brasil.; 2 Universidade do Estado do Amazonas, Manaus, AM, Brasil.; 3 Instituto Nacional de Pesquisas da Amazônia, Manaus, AM, Brasil.; 4 Instituto Todos Pela Saúde, São Paulo, SP, Brasil.; 5 Faculdade de Medicina de São José do Rio Preto, São José do Rio Preto, SP, Brasil.; 6 Universidade de Campinas, Campinas, SP, Brasil.; 7 New Mexico State University, Las Cruces, New Mexico, United States of America.; 8 University of Texas Medical Branch, Department of Pathology, Galveston, Texas, United States of America.; 9 University of Texas Medical Branch, Center for Vector-Borne and Zoonotic Diseases, Galveston, Texas, United States of America.; 10 Fundação Oswaldo Cruz Amazônia, Instituto Leônidas & Maria Deane Institute, Manaus, AM, Brasil.; 11 Universidade Nilton Lins, Manaus, AM, Brasil.

**Keywords:** Arboviruses, Surveillance, Acute febrile illness, Amazon, Brazil

## Abstract

**Background:**

Arboviral infections continue to be a significant public health challenge in the Brazilian Amazon. Overlapping symptoms, limited laboratory access, and the circulation of multiple arboviruses hamper clinical diagnosis. This study aimed to characterize the epidemiological, clinical, laboratory and genomic profiles of arboviral infections in febrile patients in Manaus, Brazil, and explore additional viral agents using metagenomic sequencing.

**Methods:**

A cross-sectional study was conducted between February 2021 and February 2023 at a tertiary reference center in Manaus, Brazil. Patients aged ≥ 5 years of age presenting with a rash and either a fever or a history of fever lasting <7 days and a negative thick blood smear for malaria were enrolled. Serum samples were tested for dengue virus (DENV), Zika virus (ZIKV), Chikungunya virus (CHIKV), yellow fever virus (YF), Oropouche virus (OROV), and Mayaro virus (MAYV) using ELISA and RT-qPCR. Positive samples were subjected to amplicon-based genome sequencing for phylogenetic analysis. A subset of RT-qPCR negative samples was analyzed using *de novo* shotgun metagenomic sequencing.

**Results:**

Among the 708 enrolled participants, 243 (34.3%) had a laboratory-confirmed arboviral infection: 92 (37.9%) DENV, 64 (26.3%) CHIKV, and 4 (1.6%) ZIKV, while 83 (34.2%) laboratory profiles were compatible with coinfection, predominantly DENV+CHIKV (55/83; 66.3%). Circulation of DENV-1 genotype V and DENV-2 genotypes III (Asian American) and II (Cosmopolitan) was identified. Metagenomic analysis of 35 samples detected *Pegivirus hominis* and *Erythroparvovirus primate* 1.

**Conclusions:**

These findings demonstrate complex arbovirus co-circulation in Manaus and support integrated surveillance strategies combining molecular, serological, and genomic approaches.

## INTRODUCTION

Arboviral diseases such as dengue, Zika, and chikungunya, caused respectively by dengue virus (DENV), Zika virus (ZIKV), and chikungunya virus (CHIKV), remain a major public health challenge in tropical and subtropical regions, since ecological and social conditions favor the proliferation of *Aedes aegypti* and *Aedes albopictus* ^1^ ^,^ ^2^ *.* Recurrent outbreaks of arboviral infections have occurred in the Americas over the past few decades, contributing to the burden of acute febrile diseases^3^ ^-^ ^5^. Brazil, particularly its Amazon region, presents favorable conditions for the circulation and maintenance of arboviruses due to its climate, widespread vector distribution, intense human mobility, and limited access to timely diagnostic resources^6^.

DENV circulates endemically throughout Brazil, with the sustained presence of all four serotypes^7^ ^-^ ^10^, while the introduction of CHIKV and the emergence of ZIKV further increased the complexity of arbovirus transmission dynamics^11^ ^,^ ^12^. Furthermore, the Oropouche virus (OROV), an orthobunyavirus frequently reported in the Amazon region, has gained prominence as a significant cause of acute fever cases. Its increasing presence further highlights the complexity of the arbovirus transmission scenario in northern Brazil^13^ ^-^ ^15^. These viruses co-circulate nationwide and frequently produce overlapping clinical manifestations and, in some cases, coinfections, which complicate diagnosis and can lead to underestimation of incidence^12^ ^,^ ^16^ ^,^ ^17^. To address this, laboratory surveillance using molecular and serological methods is necessary to complement clinical assessment, accurately identify circulating viral pathogens, estimate outbreaks, and establish monitoring and control measures^18^ ^-^ ^20^. Genomic surveillance is relevant for monitoring viral diversity, detecting new introductions, and characterizing the arbovirus transmission at regional and international levels^21^.

Manaus, capital of the Amazonas state in the northern region of Brazil, has an estimated population of approximately 2.3 million inhabitants^22^. There have been marked fluctuations in the incidence of arboviruses over the last decade, with sustained transmission of DENV, CHIKV and ZIKV^23^ ^-^ ^26^. Integrated surveillance combining clinical, laboratory, and genomic data remains limited. To this end, non-specific metagenomic tools can complement these approaches by identifying viruses not typically screened for, contributing to a comprehensive picture of viral spread. This study aimed to analyze the epidemiological, clinical, laboratory and genomic aspects of arboviral infections in febrile patients in Manaus, Brazil, and, in an exploratory manner, identify additional viral agents using metagenomic sequencing.

## METHODS

### Ethics Statement

This study was approved by the Ethics Committee of the Fundação de Medicina Tropical Doutor Heitor Vieira Dourado (FMT-HVD), CAAE:67595417.0.0000.0005, approved on November 24, 2017). It is in accordance with the Declaration of Helsinki. All participants provided written informed consent or assent.

### Study design and setting

The study was a cross-sectional investigation conducted at the Malaria Outpatient Clinic of FMT-HVD in Manaus, Brazil, between February 2021 and February 2023, a national and international reference center for tropical disease care and febrile syndromes^27^.

### Study population and sample collection

Patients aged ≥5 years were eligible if they had a fever or a recent fever lasting <7 days, a negative thick blood smear for malaria at presentation, and at least one symptom compatible with arboviral infection. Participants were included in the study during a clinical consultation at the outpatient clinic. At that time, clinical and epidemiological data were recorded, and blood samples were collected for laboratory analysis. Each participant was included only once during the acute febrile episode. Re-enrollment of the same individual in the study during the same illness episode was not permitted.

Following this initial consultation, participants were followed longitudinally, with visits scheduled on days 28 and 270 after inclusion. However, the analyses presented in this study were restricted to clinical and laboratory data collected during the initial consultation, which corresponded to the acute phase of illness.

A 5-10 mL venous blood sample was collected for serology and molecular analysis. Arboviral infections were defined based on laboratory evidence obtained through molecular and/or serological testing. Possible coinfections were defined by molecular detection and/or serological evidence of more than one arbovirus; due to flavivirus cross-reactivity, DENV- and ZIKV-IgM-positive cases without molecular confirmation were classified as FLAV.

### Laboratory diagnosis

Blood samples collected during the acute phase, at the time of participant inclusion, were subjected to serological analysis and molecular screening (RT-qPCR) for arboviruses. RT-qPCR assays were performed on these samples to detect DENV, ZIKV, CHIKV, OROV, and yellow fever (YFV).

### Serological investigation

Serum samples were screened for specific IgM antibodies against DENV, ZIKV, and CHIKV using commercially available enzyme-linked immunosorbent assay (ELISA) kits according to the manufacturers' instructions. SERION ELISA Classic Dengue Virus IgM kits were used for DENV detection, Novagnost Zika Virus IgM µ-capture for ZIKV detection, and the Euroimmun Anti-Chikungunya Virus ELISA for CHIKV detection. For each essay, serum samples were diluted according to the protocols provided by the respective kits. All necessary controls, including positive, negative, and calibrators/cut-off controls, were included on each plate. Results were interpreted qualitatively based on the manufacturer’s cutoff values. Equivocal or borderline results were retested, and if they remained equivocal, they were classified as inconclusive. 

### Nucleic acid extraction and molecular investigation

A total of 100 µL of each serum sample was submitted to RNA extraction using the Extracta kit fast DNA and RNA viral (MVXA-P096 FAST; Loccus, Brazil) according to the manufacturer’s instructions, utilizing an Extracta 96 - DNA and RNA extractor and purifier (Loccus, Brazil). A negative template control was included in each extraction batch. One-step real-time polymerase chain reaction (RT-qPCR) was performed using the GoTaq 1-Step RT-qPCR System (Promega, USA) and oligos targeting the four DENV serotypes (DENV1-4), ZIKV, CHIKV, YFV, OROV and MAYV (Table S1)^28^ ^-^ ^31^. Reactions were run on a real-time PCR platform with appropriate positive and negative controls, and samples with Ct values ≤38 were considered positive^30^ ^,^ ^31^. Positive and negative (nuclease-free water) template controls were used.

### Amplicon-based sequencing

DENV-positive samples by RT-qPCR sufficient viral load were selected for amplicon-based sequencing. The cDNA synthesis, DENV genome amplification, and library preparation were carried out using the Illumina COVIDSeq Test (Illumina, USA) modified by replacing primers as part of the kit for the primer pools primer panel for DENV-1 and DENV-2 designed by the CADDE (Brazil-UK Centre for Arbovirus Discovery, Diagnosis, Genomics and Epidemiology) project (the generated sequences from the positive DENV-1 and DENV-2 samples, Tables S2 and Table S3). Each sequencing run included a negative (nuclease-free water) and a negative template control during cDNA synthesis and amplicon generation. Library quantification was performed using the Qubit dsDNA HS assay kit on a Qubit 2.0 fluorometer (Thermo Fisher Scientific, USA). Library quality was verified using a High Sensitivity D1000 ScreenTape kit on the 4150 TapeStation system (Agilent Technologies, USA). PhiX Control v3 was used as quality control and validation for sequencing runs. Pooled libraries were normalized to 4 nM, denatured with 0.2 N NaOH, and sequenced on the Illumina MiSeq System (Illumina, USA), using a MiSeq reagent kit v2 (2 x 150 bp cycles) (Illumina, USA) with a paired-end strategy. 

### Genome assembly and consensus generation

Strict quality control of the raw demultiplexed sequencing reads was performed using Trimmomatic (v.0.39)^32^, which removed low-quality bases (minimum Phred score of Q30), sequencing adapters, primer sequences and short reads (<50 nucleotides). The cleaned paired-end reads were mapped to the DENV-1 or DENV-2 reference genome sequences (NCBI accession numbers NC_001477 for DENV-1 and NC_001474 for DENV-2) using BWA (v.0.7.17)^33^. BAM files were sorted, indexed and used for consensus sequence generation with SAMtools (v.1.10) and iVar (v.1.3.1)^34^ ^,^ ^35^.

### Shotgun sequencing and bioinformatic analysis of metagenomic data

To enhance viral surveillance, RT-qPCR-negative samples with sufficient nucleic acid concentration were selected for shotgun metagenomic sequencing. Total nucleic acids (DNA and RNA) were extracted using the MagMAX™ CORE Nucleic Acid Purification kit (Thermo Fisher Scientific, USA), following the manufacturer’s protocol. Negative extraction controls were included throughout the process to monitor potential contamination. RNA was reverse-transcribed into complementary DNA (cDNA) using a 15-nucleotide degenerate random primer (15N), enabling broad-spectrum detection of RNA viruses without prior sequence information. 

Metagenomic libraries were prepared using the Illumina^®^ DNA Prep kit (Illumina, USA). Library concentrations were quantified using the Qubit dsDNA High Sensitivity assay kit on a Qubit 2.0 fluorometer (Thermo Fisher Scientific, USA), and library quality was assessed via the High Sensitivity D1000 ScreenTape kit on the 4150 TapeStation system (Agilent Technologies, USA). Libraries were normalized to equimolar concentrations (4 nM), pooled, and sequenced on the NextSeq 1000 platform (Illumina, USA) using a P1 flow cell with paired-end 2 × 150 bp reads.

Raw FASTQ files were screened using the Chan Zuckerberg ID (CZID). Based on the taxonomic classification results, the raw sequencing data were then imported into Geneious Prime (v.2025.0.2). Reads were trimmed to remove low-quality bases and sequencing adapters, retaining only those with an average Phred quality score ≥ Q30 and a minimum length of 50 nucleotides. The resulting high-quality reads were mapped to the reference genomes of the viral taxa identified by CZID, enabling targeted reconstruction and refinement of viral sequences (NCBI accession numbers: ON340918.1 for *Pegivirus hominis* and NC_000883.2 for *Erythroparvovirus primate* 1). Consensus sequences were generated from the mapped alignments and analyzed using Genome Detective^36^.

### Phylogenetic analysis

Consensus nucleotide sequences were submitted to Genome Detective for DENV-1 and 2 genotype assignment^36^. For the phylogenetic analysis, the newly generated sequences (Table S3) were combined with DENV-1-2 complete genomes with high coverage (≤ 5 % of Ns), date, and country of collection retrieved from the EpiArbo, GISAID database (https://gisaid.org) up to July 2025^37^. The generated sequences were submitted to genotyping, which was performed using a dengue virus typing tool (Tables S4 and Table S5). To investigate the phylodynamics of DENV-1 and DENV-2, we combined the newly generated sequences with publicly available DENV-1 and DENV-2 genome sequences (Tables S6, Table S7 and Table S8). 

Phylogenetic analyses of *Pegivirus hominis* and *Erythroparvovirus primate* 1 were performed using the newly generated sequences, together with representative reference sequences retrieved from GenBank (https://www.ncbi.nlm.nih.gov/genbank)(Tables S9,S10,S11 and S12).

The nucleotide sequences were aligned using MAFFT (v.7.526)^38^ and manually curated in AliView (v.1.28)^39^. A maximum likelihood (ML) phylogenetic tree was inferred using IQ-TREE (v.1.6.12)^40^ under the best-fit substitution model implied by the ModelFinder^41^ model-selection method implemented in IQ-TREE^40^. The reliability of branching patterns was tested using the ultrafast bootstrap approximation (UFBoot), with 1,000 bootstrap alignments, and the SH-like Approximate Likelihood-ratio test (SH-aLRT), with 1,000 replicates^42^. The time-scaled ML phylogenetic trees were inferred using TreeTime^43^. Time-resolved trees were not generated for the *Pegivirus hominis* and *Erythroparvovirus primate* 1 datasets due to limited temporal signal and sample size. All trees were visualized and edited using R statistical software (v.4.3.1)^44^.

### Statistical analysis

Continuous variables with a normal distribution were presented as mean and standard deviation, while categorical variables were described as n/N (percentage). For exploratory comparisons between groups, ANOVA was used for continuous variables with a normal distribution, and Fisher's exact test was used for categorical variables. Given the descriptive nature of the study, the absence of pre-established hypotheses, and the lack of a formal sample size calculation, these analyses should be understood as exploratory. For this reason, the p-values presented need to be interpreted with caution, as no formal adjustment for multiple comparisons was performed. All statistical analyses were performed using R software (version 4.4.2)^44^ .

## RESULTS

### Demographic and clinical characteristics

Between February 2021 and February 2023, 708 patients with symptoms suggestive of arboviruses were recruited, and 243 (34.3%) had laboratory-confirmed infection: 92 (37.9%) were positive for DENV, 64 (26.3%) for CHIKV, and 4 (1.6%) for ZIKV, while 83 (34.2%) had serological and/or molecular profiles compatible with possible coinfection , predominantly DENV+CHIKV (55; 66.3%) (Figure S1 and Table 1). No infections caused by OROV, YFV or MAYV were detected. DENV-1 (18/243; 7.4%) and DENV-2 (3/243; 1.2%) were detected. Most cases occurred in 2022 (458; 63.4%). The distribution of infections by year is shown in Table 2.

**TABLE 1:** Clinical and laboratory characteristics by arbovirus group.

Characteristics	Overall	DENV	CHIKV	ZIKV	DENV+CHIKV	FLAV	FLAV+CHIKV	***p*-value** ^*2*^
	n = 243^1^	n= 92^1^	n= 64^1^	n = 4^1^	n = 55^1^	n = 14^1^	n = 14^1^	
**Age**	40 (16)	40 (16)	40 (15)	38 (12)	39 (17)	46 (24)	37 (13)	>0.9
**Sex:**								0.5
Male	138/243 (57%)	47/92 (51%)	38/64 (59%)	4/4 (100%)	32/55 (58%)	8/14 (57%)	9/14 (64%)	
Female	105/243 (43%)	45/92 (49%)	26/64 (41%)	0/4 (0%)	23/55 (42%)	6/14 (43%)	5/14 (36%)	
**Ethnic group:**								0.5
White	43/241 (18%)	20/92 (22%)	10/64 (16%)	0/3 (0%)	10/54 (19%)	1/14 (7.1%)	2/14 (14%)	
Black	16/241 (6.6%)	9/92 (9.8%)	2/64 (3.1%)	0/3 (0%)	3/54 (5.6%)	0/14 (0%)	2/14 (14%)	
Mixed race	168/241 (70%)	61/92 (66%)	47/64 (73%)	3/3 (100%)	35/54 (65%)	12/14 (86%)	10/14 (71%)	
Asian	2/241 (0.8%)	1/92 (1.1%)	0/64 (0%)	0/3 (0%)	1/54 (1.9%)	0/14 (0%)	0/14 (0%)	
Indigenous	12/241 (5.0%)	1/92 (1.1%)	5/64 (7.8%)	0/3 (0%)	5/54 (9.3%)	1/14 (7.1%)	0/14 (0%)	
**Comorbidities:**								
Diabetes mellitus	13/242 (5.4%)	6/91 (6.6%)	3/64 (4.7%)	0/4 (0%)	2/55 (3.6%)	2/14 (14%)	0/14 (0%)	0.7
Hypertension	24/242 (9.9%)	12/91 (13%)	3/64 (4.7%)	1/4 (25%)	6/55 (11%)	2/14 (14%)	0/14 (0%)	0.3
Coronary heart disease	3/242 (1.2%)	3/91 (3.3%)	0/64 (0%)	0/4 (0%)	0/55 (0%)	0/14 (0%)	0/14 (0%)	0.6
Hyperlipidemia	9/242 (3.7%)	8/91 (8.8%)	1/64 (1.6%)	0/4 (0%)	0/55 (0%)	0/14 (0%)	0/14 (0%)	**0.032**
Asthma	4/242 (1.7%)	2/91 (2.2%)	1/64 (1.6%)	0/4 (0%)	1/55 (1.8%)	0/14 (0%)	0/14 (0%)	>0.9
COPD	3/242 (1.2%)	3/91 (3.3%)	0/64 (0%)	0/4 (0%)	0/55 (0%)	0/14 (0%)	0/14 (0%)	0.6
TB	1/242 (0.4%)	1/91 (1.1%)	0/64 (0%)	0/4 (0%)	0/55 (0%)	0/14 (0%)	0/14 (0%)	>0.9
Known retroviral infection	2/242 (0.8%)	2/91 (2.2%)	0/64 (0%)	0/4 (0%)	0/55 (0%)	0/14 (0%)	0/14 (0%)	0.7
Renal disease	1/241 (0.4%)	1/91 (1.1%)	0/64 (0%)	0/4 (0%)	0/54 (0%)	0/14 (0%)	0/14 (0%)	>0.9
**Contact with person(s) who recently had an arbovirus infection**	24/243 (9.9%)	18/92 (20%)	1/64 (1.6%)	1/4 (25%)	4/55 (7.3%)	0/14 (0%)	0/14 (0%)	**0.002**
**Signs and symptoms**								
Fever	239/243 (98%)	88/92 (96%)	64/64 (100%)	4/4 (100%)	55/55 (100%)	14/14 (100%)	14/14 (100%)	0.3
Facial flush	51/243 (21%)	39/92 (42%)	1/64 (1.6%)	2/4 (50%)	8/55 (15%)	1/14 (7.1%)	0/14 (0%)	**<0.001**
Cutaneous hemorrhage	2/243 (0.8%)	2/92 (2.2%)	0/64 (0%)	0/4 (0%)	0/55 (0%)	0/14 (0%)	0/14 (0%)	0.6
Rashes/skin changes	75/243 (31%)	52/92 (57%)	7/64 (11%)	0/4 (0%)	12/55 (22%)	3/14 (21%)	1/14 (7.1%)	**<0.001**
Muscle pain	209/243 (86%)	84/92 (91%)	54/64 (84%)	4/4 (100%)	45/55 (82%)	10/14 (71%)	12/14 (86%)	0.3
Pain in bones and/or joints	133/243 (55%)	69/92 (75%)	25/64 (39%)	3/4 (75%)	29/55 (53%)	1/14 (7.1%)	6/14 (43%)	**<0.001**
Retro-orbital pain	105/243 (43%)	59/92 (64%)	18/64 (28%)	3/4 (75%)	20/55 (36%)	2/14 (14%)	3/14 (21%)	**<0.001**
Abdominal pain	79/243 (33%)	36/92 (39%)	18/64 (28%)	2/4 (50%)	20/55 (36%)	2/14 (14%)	1/14 (7.1%)	0.072
Vomiting	63/243 (26%)	23/92 (25%)	16/64 (25%)	0/4 (0%)	14/55 (25%)	4/14 (29%)	6/14 (43%)	0.7
Diarrhea	71/243 (29%)	34/92 (37%)	11/64 (17%)	2/4 (50%)	16/55 (29%)	5/14 (36%)	3/14 (21%)	0.094
Sore throat	49/243 (20%)	24/92 (26%)	9/64 (14%)	0/4 (0%)	16/55 (29%)	0/14 (0%)	0/14 (0%)	**0.011**
Coryza	23/243 (9.5%)	15/92 (16%)	3/64 (4.7%)	0/4 (0%)	5/55 (9.1%)	0/14 (0%)	0/14 (0%)	0.13
Conjunctivitis	25/243 (10%)	21/92 (23%)	0/64 (0%)	0/4 (0%)	4/55 (7.3%)	0/14 (0%)	0/14 (0%)	**<0.001**
Cough	42/243 (17%)	18/92 (20%)	10/64 (16%)	1/4 (25%)	11/55 (20%)	2/14 (14%)	0/14 (0%)	0.5
Shortness of breath	34/243 (14%)	20/92 (22%)	6/64 (9.4%)	2/4 (50%)	6/55 (11%)	0/14 (0%)	0/14 (0%)	**0.014**
Oliguria	27/243 (11%)	16/92 (17%)	2/64 (3.1%)	1/4 (25%)	8/55 (15%)	0/14 (0%)	0/14 (0%)	**0.017**
Jaundice	5/243 (2.1%)	1/92 (1.1%)	1/64 (1.6%)	1/4 (25%)	1/55 (1.8%)	0/14 (0%)	1/14 (7.1%)	0.081
Edema	29/243 (12%)	23/92 (25%)	2/64 (3.1%)	0/4 (0%)	4/55 (7.3%)	0/14 (0%)	0/14 (0%)	**<0.001**
Bleeding	18/239 (7.5%)	11/90 (12%)	2/63 (3.2%)	0/4 (0%)	5/54 (9.3%)	0/14 (0%)	0/14 (0%)	0.3
Gums	3/15 (20%)	1/9 (11%)	1/2 (50%)	0/0 (NA%)	1/4 (25%)	0/0 (NA%)	0/0 (NA%)	0.5
Epistaxis	3/15 (20%)	2/9 (22%)	0/2 (0%)	0/0 (NA%)	1/4 (25%)	0/0 (NA%)	0/0 (NA%)	>0.9
Hemoptysis	2/15 (13%)	2/9 (22%)	0/2 (0%)	0/0 (NA%)	0/4 (0%)	0/0 (NA%)	0/0 (NA%)	>0.9
Melena	8/15 (53%)	5/9 (56%)	0/2 (0%)	0/0 (NA%)	3/4 (75%)	0/0 (NA%)	0/0 (NA%)	0.4
Hematuria	2/15 (13%)	1/9 (11%)	0/2 (0%)	0/0 (NA%)	1/4 (25%)	0/0 (NA%)	0/0 (NA%)	>0.9
Menorrhagia	5/18 (28%)	3/11 (27%)	1/2 (50%)	0/0 (NA%)	1/5 (20%)	0/0 (NA%)	0/0 (NA%)	>0.9
Headache	233/242 (96%)	87/91 (96%)	62/64 (97%)	4/4 (100%)	53/55 (96%)	13/14 (93%)	14/14 (100%)	0.9
Choluria	27/242 (11%)	17/91 (19%)	1/64 (1.6%)	1/4 (25%)	7/55 (13%)	0/14 (0%)	1/14 (7.1%)	**0.006**
Thin skin	102/242 (42%)	35/91 (38%)	26/64 (41%)	3/4 (75%)	24/55 (44%)	5/14 (36%)	9/14 (64%)	0.4
Itching	60/242 (25%)	43/91 (47%)	3/64 (4.7%)	0/4 (0%)	13/55 (24%)	1/14 (7.1%)	0/14 (0%)	**<0.001**
Weakness of the lower limbs	138/240 (58%)	55/90 (61%)	30/64 (47%)	1/4 (25%)	36/54 (67%)	9/14 (64%)	7/14 (50%)	0.2
Lethargic state	5/242 (2.1%)	5/91 (5.5%)	0/64 (0%)	0/4 (0%)	0/55 (0%)	0/14 (0%)	0/14 (0%)	0.3
Paleness	57/238 (24%)	10/88 (11%)	17/64 (27%)	0/4 (0%)	15/54 (28%)	9/14 (64%)	6/14 (43%)	0.3
**Hydration**								**0.003**
Normal	186/241 (77%)	79/90 (88%)	48/64 (75%)	4/4 (100%)	38/55 (69%)	6/14 (43%)	11/14 (79%)	
Dehydrated	55/241 (23%)	11/90 (12%)	16/64 (25%)	0/4 (0%)	17/55 (31%)	8/14 (57%)	3/14 (21%)	
Edematous	0/241 (0%)	0/90 (0%)	0/64 (0%)	0/4 (0%)	0/55 (0%)	0/14 (0%)	0/14 (0%)	
Oropharynx injected	8/240 (3.3%)	5/90 (5.6%)	1/64 (1.6%)	0/4 (0%)	2/54 (3.7%)	0/14 (0%)	0/14 (0%)	0.8
Palate with petechia	5/242 (2.1%)	4/91 (4.4%)	0/64 (0%)	0/4 (0%)	1/55 (1.8%)	0/14 (0%)	0/14 (0%)	0.5
Lymphadenopathy	16/242 (6.6%)	11/91 (12%)	1/64 (1.6%)	0/4 (0%)	4/55 (7.3%)	0/14 (0%)	0/14 (0%)	0.14
Temperature	36.67 (0.80)	36.60 (0.85)	36.68 (0.78)	36.68 (1.85)	36.85 (0.73)	36.57 (0.76)	36.54 (0.45)	0.5
Respiratory frequency (/min)	18.95 (1.00)	18.90 (1.03)	19.02 (0.92)	19.25 (1.50)	18.91 (1.09)	19.14 (0.86)	18.86 (0.95)	>0.9
Diastolic pressure	80 (14)	80 (13)	84 (14)	81 (11)	78 (15)	76 (11)	82 (18)	0.13
Systolic pressure	120 (17)	119 (16)	125 (18)	122 (16)	119 (17)	115 (14)	120 (24)	0.12
Arterial pulse irregular	8/238 (3.4%)	2/89 (2.2%)	3/62 (4.8%)	0/4 (0%)	0/55 (0%)	1/14 (7.1%)	2/14 (14%)	0.085
Facial or skin flushing	31/242 (13%)	24/91 (26%)	1/64 (1.6%)	1/4 (25%)	5/55 (9.1%)	0/14 (0%)	0/14 (0%)	**<0.001**
Generalized skin rash	37/242 (15%)	31/91 (34%)	0/64 (0%)	0/4 (0%)	6/55 (11%)	0/14 (0%)	0/14 (0%)	**<0.001**
Mottled skin	23/241 (9.5%)	19/90 (21%)	1/64 (1.6%)	0/4 (0%)	3/55 (5.5%)	0/14 (0%)	0/14 (0%)	**<0.001**
Cold and sticky skin	13/242 (5.4%)	11/91 (12%)	0/64 (0%)	0/4 (0%)	2/55 (3.6%)	0/14 (0%)	0/14 (0%)	**0.025**
Auscultation abnormal	3/239 (1.3%)	3/91 (3.3%)	0/63 (0%)	0/4 (0%)	0/53 (0%)	0/14 (0%)	0/14 (0%)	0.5
**Laboratory characteristics**								
Red cells	4.88 (0.63)	4.97 (0.55)	4.98 (0.60)	5.39 (0.71)	4.68 (0.71)	4.75 (0.77)	4.65 (0.45)	0.03
Hemoglobin	14.14 (1.82)	14.47 (1.76)	14.46 (1.54)	15.25 (1.55)	13.47 (2.00)	13.73 (2.01)	13.29 (1.85)	**0.006**
Hematocrit	42.2 (5.3)	43.5 (5.0)	42.6 (4.5)	45.7 (4.6)	40.3 (5.8)	41.7 (6.1)	39.4 (4.9)	**0.003**
MCV	86.5 (5.5)	87.7 (5.0)	85.8 (5.0)	85.0 (3.3)	85.6 (5.2)	88.5 (9.4)	84.8 (5.3)	**0.013**
MCH	29.02 (2.26)	29.15 (1.87)	29.19 (2.27)	28.39 (0.84)	28.73 (2.49)	29.16 (3.30)	28.60 (2.81)	0.5
MCHC	33.52 (1.39)	33.27 (1.14)	33.98 (1.41)	33.41 (0.77)	33.48 (1.58)	32.95 (0.80)	33.68 (2.06)	**0.005**
RDW	11.89 (1.30)	11.82 (0.99)	11.54 (1.09)	11.85 (0.76)	12.12 (1.27)	12.33 (2.19)	12.65 (2.24)	0.05
Leukocytes	6,200 (4,787)	5,221 (3,498)	6,426 (2,157)	10,522 (4,525)	7,341 (8,182)	6,291 (4,206)	5,617 (2,275)	**<0.001**
Segmented	58 (16)	54 (17)	61 (15)	67 (13)	59 (15)	60 (18)	59 (17)	0.2
Eosinophils	2.50 (3.33)	2.54 (3.03)	2.50 (3.19)	1.25 (1.08)	2.81 (4.59)	1.99 (1.23)	2.04 (1.82)	0.9
Basophils	0.45 (0.64)	0.55 (0.86)	0.41 (0.48)	0.45 (0.54)	0.43 (0.50)	0.26 (0.38)	0.36 (0.30)	>0.9
Lymphocytes	30 (15)	33 (16)	28 (13)	24 (12)	28 (13)	28 (17)	30 (17)	0.3
Monocytes	9.0 (4.0)	9.5 (4.3)	8.0 (3.5)	7.0 (1.5)	9.7 (4.3)	8.5 (2.8)	8.7 (4.2)	0.11
Platelet count	172,347 (95,986)	163,686 (86,836)	181,198 (87,324)	358,725 (185,002)	163,018 (97,439)	177,574 (88,298)	161,268 (117,697)	**0.034**
MPV	7.75 (1.81)	7.92 (1.76)	7.42 (1.67)	6.78 (1.50)	7.93 (1.96)	8.03 (1.87)	7.47 (2.07)	0.3
PCT	0.12 (0.05)	0.12 (0.04)	0.13 (0.05)	0.22 (0.06)	0.12 (0.06)	0.14 (0.06)	0.10 (0.06)	**0.009**
PDW	29.26 (136.72)	44.73 (224.38)	19.79 (2.91)	19.63 (1.64)	20.30 (1.51)	20.86 (1.57)	20.47 (1.96)	0.4

^1^Mean (SD); n/N (%), ^2^Fisher’s exact test. **MCV:** Mean corpuscular volume, **MCH:** Mean corpuscular hemoglobin, **MCHC:** Mean corpuscular hemoglobin concentration, **RDW:** Red cell distribution width, **MPV:** Mean platelet volume, **PCT:** Plateletcrit, **PDW:** Platelet distribution width.

**TABLE 2:** Distribution of cases over the years.

	Overall	2021	2022	2023
	n = 708^*1*^	n= 77^*1*^	n = 458^*1*^	n = 173^1^
**Positivity**				
DENV	92/708 (13%)	31/77 (40%)	53/458 (12%)	8/173 (4.6%)
CHIKV	64/708 (9.0%)	0/77 (0%)	43/458 (9.4%)	21/173 (12%)
ZIKV	4/708 (0.6%)	1/77 (1.3%)	3/458 (0.7%)	0/173 (0%)
DENV+CHIKV	55/708 (7.8%)	3/77 (3.9%)	43/458 (9.4%)	9/173 (5.2%)
FLAV	14/708 (2.0%)	0/77 (0%)	6/458 (1.3%)	8/173 (4.6%)
ZIKV+CHIKV	0/708 (0%)	0/77 (0%)	0/458 (0%)	0/173 (0%)
FLAV+CHIKV	14/708 (2.0%)	0/77 (0%)	6/458 (1.3%)	8/173 (4.6%)
**ELISA DENV IgM:**				
Positive	158/708 (22%)	31/77 (40%)	98/458 (21%)	29/173 (17%)
Negative	405/708 (57%)	39/77 (51%)	256/458 (56%)	110/173 (64%)
Indeterminate	145/708 (20%)	7/77 (9.1%)	104/458 (23%)	34/173 (20%)
**PCR DENV**				
Positive	21/708 (3.0%)	3/77 (3.9%)	13/458 (2.8%)	5/173 (2.9%)
Negative	687/708 (97%)	74/77 (96%)	445/458 (97%)	168/173 (97%)
**ELISA ZIKV IgM**				
Positive	32/708 (4.5%)	1/77 (1.3%)	15/458 (3.3%)	16/173 (9.2%)
Negative	609/708 (86%)	62/77 (81%)	400/458 (87%)	147/173 (85%)
Indeterminate	67/708 (9.5%)	14/77 (18%)	43/458 (9.4%)	10/173 (5.8%)
**ELISA CHICK IgM**				
Positive	133/708 (19%)	3/77 (3.9%)	92/458 (20%)	38/173 (22%)
Negative	500/708 (71%)	66/77 (86%)	322/458 (70%)	112/173 (65%)
Indeterminate	75/708 (11%)	8/77 (10%)	44/458 (9.6%)	23/173 (13%)

^1^ n/N (%).

Most participants were men (138; 57%), with a mean age of 40 years, and were of mixed race (168; 70%) (Table 1). The most frequent comorbidity was hypertension (24; 9.9%), with a difference for hyperlipidemia, which was prevalent in DENV infection (8; 8.8%). Previous contact with individuals with recent arbovirus infections was common among patients with DENV (18; 20%) and ZIKV (1; 25%).

Facial flushing (39; 42%), rash/skin changes (52; 57%), generalized rash (31; 34%), conjunctivitis (21; 23%), pruritus (43; 47%), peripheral edema (23; 25%), mottled skin (19; 21%), and cold and clammy skin (11; 12%) were frequently observed among individuals with DENV infection. The overall analysis indicated a difference in the distribution of these clinical manifestations across all study groups (*p* < 0.005). Bone and/or joint pain (69; 75% in DENV; 29; 53% in DENV+CHIKV) and retro-orbital pain (59; 64% in DENV; 20; 36% in DENV+CHIKV), as well as sore throat (24; 26% in DENV; 16; 29% in DENV+CHIKV), dyspnea (20; 22% in DENV; 6; 11% in DENV+CHIKV), oliguria (16; 17% in DENV; 8; 15% in DENV+CHIKV), and choluria (17; 19% in DENV; 7; 13% in DENV+CHIKV), showed variation in frequency among the groups. Exploratory analysis indicated differences in symptom distribution across groups (p ≤ 0.017).. Differences in hydration status were observed in the global comparison, with a higher proportion of dehydration observed in individuals infected with CHIKV (16; 25%) and those with DENV+CHIKV coinfection (17; 31%) (*p* = 0.003).

Regarding laboratory parameters, lower erythrocyte, hemoglobin, and hematocrit levels were observed in coinfections (DENV+CHIKV; erythrocytes: 4.68-4.75 million/µL; hemoglobin: 13.29-13.73 g/dL; hematocrit: 39.4-41.7% and FLAV+CHIKV; erythrocytes: 4.68-4.75 million/µL; hemoglobin: 13.29-13.73 g/dL; hematocrit: 39.4-41.7%) compared with the other groups. The statistical analysis indicated differences across groups for erythrocytes (*p* = 0.03), hemoglobin (*p* = 0.006), and hematocrit (*p* = 0.003), based on global tests only. Leukocyte levels were lowest in the DENV group (5,221/µL), with difference across groups (*p* < 0.001). Platelet counts varied among groups, being lower in the DENV/FLAV group (163,018-177,574/µL) and higher in the FLAV group, with an overall group effect (*p* = 0.034). Plateletcrit was also higher in the CHIKV group, with a global difference across groups (*p* = 0.009). Mean corpuscular volume showed moderate variation among groups (CHIKV and DENV+CHIKV: 85.0-85.8 fL; FLAV: 88.5 fL), with an overall difference (*p* = 0.013). Mean corpuscular hemoglobin concentration (MCHC) showed minimal variation (32.95-33.98 g/dL), although a global difference across groups was detected (*p* = 0.005). Red Cell Distribution Width values were higher in the FLAV and FLAV+CHIKV groups (12.33-12.65%) compared with the DENV and CHIKV groups (11.54-11.89%), with a marginal difference across groups (*p* = 0.05).

### Phylodynamics of DENV

**FIGURE 1:**
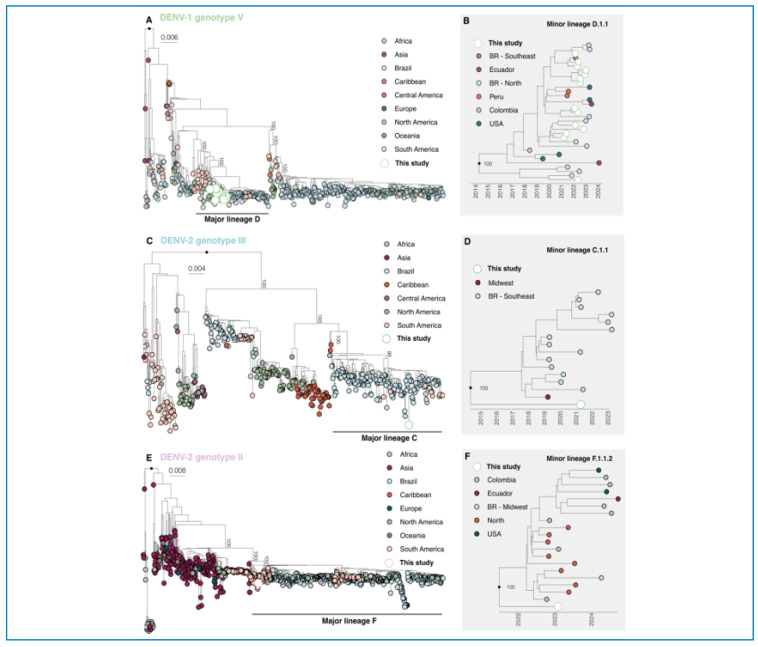
**Phylogenetic analysis of DENV in Manaus 2021-2023. (A)** Maximum likelihood (ML) phylogenetic analysis of 14 complete genome sequences from DENV-1 genotype V (DENV-1-GV) generated in this study (2021-2023), plus 549 publicly available sequences. The sequences grouped within the major clade E. **(B)** Time-scaled DENV-1-GV phylogenetic subtree of major clade E, minor lineage D.1.1. **(C)** ML phylogenetic analysis of one (2021) complete genome sequences from DENV-2 genotype III (Asian American) (DENV-2-GIII) generated in this study, plus 482 publicly available sequences. The sequence is grouped within the major clade C. **(D)** Time-scaled DENV-2-GIII phylogenetic subtree of major clade C, minor lineage C.1.1. **(E)** ML phylogenetic analysis of one (2023) complete genome sequence from DENV-2 genotype II (Cosmopolitan) (DENV-2-GII) generated in this study, plus 1,005 publicly available sequences. The sequence is grouped within the major clade F. (F) Time-scaled DENV-2-GII phylogenetic subtree of major clade F, minor lineage F.1.1.2. The trees are mid-point rooted, and the scale bar is in units of nucleotide substitutions per site (s/s). Colors of the dots represent different sampling locations and genome sequences generated in this study.

The newly generated DENV-1 sequences were classified as belonging to genotype V (DENV-1-V), while DENV-2 sequences were classified into genotypes III (Asian American) and II (Cosmopolitan) (Figures 1A, 1C and 1E). Phylogenetic analysis revealed that the generated sequences clustered within the major lineage D and the minor lineage D.1.1 (Figures 1A and 1B). The DENV-GIII sequence was found within clade C, specifically minor lineage C.1.1 (Figures 1C and 1D), Phylogenetic analyses revealed clustering within lineages closely related to strains circulating in South America and multiple regions of Brazil, suggesting regional and interregional viral dispersion (Figure 1; Tables S4 and Table S5).

### Metagenomic analyses

**FIGURE 2:**
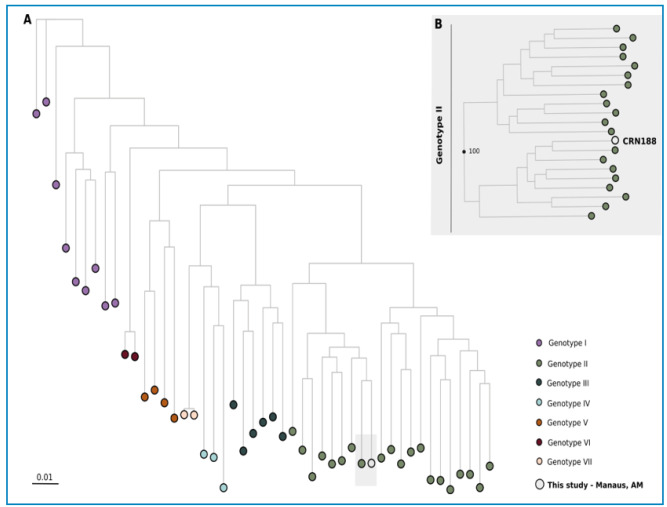
**Phylogenetic analysis of Pegivirus hominis in Manaus. (A)** Maximum likelihood (ML) phylogenetic tree based on an 8,988-nucleotide coding region of the *Pegivirus hominis* genome. The dataset comprises 47 sequences, including one sequence generated in this study (Manaus, Brazil, 2022) and 46 reference sequences retrieved from GenBank, representing different genotypes. **(B)** Enlarged view of the clade containing the one sequence from this study (CRN188), which clustered within Genotype II.

**FIGURE 3:**
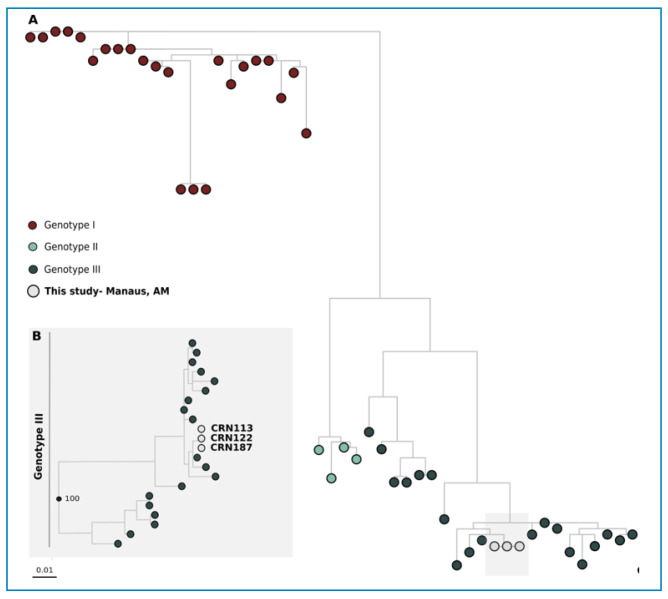
**Phylogenetic analysis of Erythroparvovirus primate 1 in Manaus. (A)** Maximum likelihood (ML) phylogenetic tree based on a 423-nucleotide fragment of the partial VP1/VP2 coding region of *Erythroparvovirus primate 1*. The dataset includes three sequences obtained in this study (Manaus, Brazil, 2022) and 50 reference sequences retrieved from GenBank. Sequences are grouped by genotype, represented by distinct colors. The sequences from Manaus clustered within Genotype III. **(B)** Detail of the clade corresponding to Genotype III, showing the placement of the three sequences generated in this study (CRN113, CRN122 and CRN187).

Shotgun metagenomic sequencing of 35 serum samples identified DENV-1 in two cases, both clustering within genotype V and confirmed by serotype-specific RT-PCR (Figure 1). In addition, *Pegivirus hominis* (genotype II in 2 samples, Figure 2; Table S10) and *Erythroparvovirus primate 1* (genotype III, Figure 3; Table S11) were detected in a subset of samples and confirmed through phylogenetic analysis (Figure 2 and Figure 3).

## DISCUSSION

This cross-sectional study examined the circulation of ZIKV, DENV, CHIKV, OROV, YFV, and MAYV among patients with suspected arboviral infection attending a tertiary healthcare unit in Manaus between February 2021 and February 2023. Infections with DENV, CHIKV, and ZIKV were confirmed using serological and molecular techniques, with DENV predominating; infections with *Erythroparvovirus primate* 1 and, probably incidentally, *Pegivirus hominis* were also identified. Furthermore, serological profiles suggest potential coinfections; these findings should be interpreted cautiously, as they do not constitute definitive evidence of simultaneous infection. A marked increase in arboviral cases was observed in 2022, coinciding with reports of increased OROV activity in the region, coinciding^45^. These findings highlight the dynamic nature of arbovirus circulation and the need for continuous surveillance. Although essential, laboratory surveillance of arboviruses in Brazil is primarily limited to scientific research and academic initiatives.

Manaus has historically faced recurring dengue outbreaks, characterized by the sequential emergence and co-circulation of multiple DENV serotypes^23^ ^,^ ^46^ ^-^ ^48^. Detection of DENV-1 and DENV-2 aligns with previous regional reports. Frequent laboratory profiles indicating possible DENV+CHIKV coinfections support findings from other studies in the Brazilian^49^ ^-^ ^51^, and highlight the diagnostic challenges in endemic areas. The presence of probable coinfections poses an additional challenge for both diagnosis and clinical management, which may be associated with more pronounced hematological alterations.

OROV is considered an important cause of acute febrile illnesses in the Amazon region^13^ ^,^ ^52^. In recent years, especially between 2023 and 2024, OROV has regained epidemiological prominence due to the expansion of outbreaks recorded not only in Brazil but also in other parts of South America^53^ ^-^ ^56^. Even after submitting all acute-phase samples from participants to RT-qPCR screening for OROV, no positive results were identified. The absence of detections may be associated with temporal fluctuations in viral circulation, differences inherent to local transmission dynamics, or the epidemiological context of the study period. Nevertheless, our findings reinforce the need to maintain broad molecular surveillance strategies for arboviruses in the Amazon, considering that several pathogens can circulate simultaneously and contribute in different ways to acute fever in the region^13^ ^-^ ^15^.

Regarding signs and symptoms, while overlap among arboviruses, DENV infections frequently display cutaneous and vasomotor signs, consistent with classic dengue^57^ ^-^ ^59^. Bone and joint pain, as well as retro-orbital pain, are common in both DENV infections and cases with laboratory evidence of possible DENV and CHIKV coinfection. Both viruses trigger a strong systemic inflammatory response, with CHIKV inducing a more pronounced reaction^60^ ^-^ ^62^. Although some signs may aid clinical suspicion, the overlap in symptoms limits the value of clinical features alone, underscoring the need for combined serological and molecular testing. 

Laboratory parameters vary depending on the virus causing the infection. DENV+CHIKV and FLAV+CHIKV showed reductions in erythrocyte, hemoglobin, and hematocrit levels. Experimental studies involving arbovirus coinfections demonstrate substantial reductions in red blood cell counts and hematocrit values during the acute phase. These alterations are consistent with intense systemic inflammatory responses and possible impairment of erythropoiesis in coinfections^63^. These results are consistent with research that points to a possible immune dysregulation during arbovirus co-infections^64^. However, due to the cross-sectional design of this study, it is not possible to establish cause-and-effect relationships between the observed phenomena. However, these findings should be interpreted with caution, given the study’s cross-sectional design and the absence of data on clinical outcomes. Leukopenia and thrombocytopenia, typical of DENV infection, were confirmed, while the reduction in platelets in the DENV+FLAV group suggests a combined effect on their consumption or destruction. The increase in plateletcrit in CHIKV cases may reflect a compensatory response or platelet activation associated with the CHIKV-induced inflammatory process. 

Phylogenetic analyses revealed the co-circulation of different DENV-1 and DENV-2 genotypes in Manaus, with a predominance of DENV-1 genotype V and detection of DENV-2 genotypes II (Cosmopolitan) and III (Asian American). The circulation of DENV-1-V in Manaus is consistent with patterns observed in recent years in other regions of Brazil^65^. The origin of DENV-1-V is traced to the Caribbean and Venezuela; however, multiple independent introductions over time have led to high genetic variability and intense viral exchange in South America, with the Brazilian Amazon potentially acting as a regional connectivity hub^57^ ^-^ ^60^. The clustering of samples from Manaus with sequences from the southeast and north of Brazil reinforces the notion that the country is nationally interconnected in terms of viral dissemination, raising concerns about the speed at which new genomic variants may spread in large urban centers and peripheral areas. 

In Brazil, DENV-2 genotype III was first detected in 1990, followed by additional introductions through 2022, primarily from Caribbean countries and northern South America^66^. In the Amazon, genotype III was first reported in Acre in 2021^67^ ^,^ ^68^. Its introduction into the state of Amazonas likely occurred between May 2020 and August 2022^66^. In the present study, DENV-2 genotype II sequences clustered within clade F, sublineage F.1.2, and are closely related to sequences from several South American countries, consistent with regional dispersal. In contrast, the identification of a DENV-2 genotype III sequence within clade C, sublineage C.1.1, is consistent with intranational dissemination, likely originating in the northern region of Brazil and subsequently expanding to the southeastern and midwestern regions. This may indicate the Amazon region as an important area for the emergence and spread of viral lineages.

Metagenomic analysis of 35 serum samples identified *Pegivirus hominis* and *Erythroparvovirus primate* 1. The detection of *Pegivirus hominis* reflects an incidental finding due to the high sensitivity of metagenomic techniques, rather than a causal relationship with the clinical presentation^61^ ^-^ ^63^. Although its direct pathogenicity has not been established, the identification of this virus underscores the importance of non-targeted approaches to laboratory surveillance of febrile syndromes in the Amazon, which may reveal the diversity of circulating viruses and their potential interactions with endemic infections such as dengue and other arboviruses. Further studies with larger samples and improved sequencing are needed to determine the clinical significance of this virus. Analysis of the viral sequences placed the isolate within genotype 2, which is well-documented in Brazil^69^ ^-^ ^77^. 

The detection of *Erythroparvovirus primate* 1 in three serum samples, all belonging to genotype III, suggests the circulation of a specific lineage in the studied population. Genotype 3 of this species appeared to be restricted to Africa but has already been detected in countries outside this continent, including Brazil^78^ ^-^ ^80^. Although parvovirus *Erythroparvovirus primate 1* is not typically considered a febrile illness, fever may occur in some cases. The clinical presentation is variable and often nonspecific, making clinical suspicion difficult and contributing to underdiagnosis. The low frequency observed limits inferences on the magnitude of local transmission. The finding highlights the importance of molecular surveillance, particularly because viremia is typically short-lived and may be underestimated in serum samples.

This study has limitations. First, the cross-sectional design of this study, coupled with the fact that the clinical and laboratory data analyzed were collected during a single consultation, limits the ability to monitor symptom evolution and laboratory changes throughout the course of the infection. Although the protocol provided for follow-up visits, longitudinal data were not considered in the analysis presented here. Furthermore, the selection of participants from a tertiary referral center may have introduced selection biases. More restrictive clinical inclusion criteria and the likelihood of low viremia at the time of collection may have reduced case detection, while cross-reactivity observed in flavivirus serology may have affected the correct classification of infections. The absence of clinical follow-up prevented confirmation of recent infections, and metagenomic analyses limited to a subset of samples likely underestimated viral diversity. Furthermore, arbovirus transmission patterns may have been influenced by the COVID-19 pandemic. These limitations reinforce the need for further longitudinal studies with integrated approaches to better understand the dynamics of these infections.

In conclusion, this study highlights the circulation of ZIKV, CHIKV, and DENV in Manaus from 2021 to 2023 and underscores the importance of integrated surveillance across clinical, molecular, and serological dimensions. The application of metagenomic strategies revealed that febrile syndromes in the Amazon may be associated with viruses other than known arboviruses, suggesting viral diversity that may not yet be recognized. Phylogenetic analysis helped track the current and past circulation of these viruses in the region. Future studies should focus on coinfections, their clinical management and associated clinical outcomes, and the role of emerging or re-emerging viruses in the profile of febrile illnesses in the Amazon.

## Data Availability

The data is available for access at https://doi.org/10.5281/zenodo.18321098. Sequences and sequencing data generated in this study have been deposited in GISAID and GenBank databases, under the accession numbers listed in Supplementary Tables 4, 10 and 11
