# Supplementary material for: Integrated surveillance of arboviruses in febrile patients from the Brazilian Amazon reveals complex co-circulation dynamics and hidden viral diversity
Source: Rev Soc Bras Med Trop. 2026 Jul 17;59(Suppl 1):e0042-2026. doi: 10.1590/0037-8682-0042-2026 (PMC13379192; doi:10.1590/0037-8682-0042-2026)
Supplement: Supplementary material [file 1678-9849-rsbmt-59-s1-e0042-2026-md1.pdf]

Supplementary Table 1. Primers and probes for dengue virus (DENV1-4), chikungunya virus (CHIKV), Zika virus (ZIKV), yellow fever virus (YFV), Oropouche virus (OROV), and Mayaro virus (MAYV) detection using RT-qPCR.

Primer/Probe	Sequence (5'-3')	Target
DEN-1 F	CAAAAGGAAGTCGTGCAATA	NS5
DEN-1 C	CTGAGTGAATTCTCTCTACTGAACC	
DEN-1 P	FAM-CATGTGGTTGGGAGCACGC-BHQ1	
DEN-2 F	CAGGTTATGGCACTGTCACGAT	E
DEN-2 C	CCATCTGCAGCAACACCATCTC	
DEN-2 P	HEX-CTCTCCGAGAACAGGCCTCGACTTCAA-BHQ1	
DEN-3 F	GGACTGGACACACGCACTCA	prM
DEN-3 C	CATGTCTCTACCTTCTCGACTTGTCT	
DEN-3 P	TAMRA-ACCTGGATGTCGGCTGAAGGAGCTTG-BHQ2	
DEN-4 F	TTGTCCTAATGATGCTGGTCG	prM/E
DEN-4 C	TCCACCTGAGACTCCTTCCA	
DEN-4 P	TexasRed-TTCCTACTCCTACGCATCGCATTCCG-BHQ3	
DENV F	TAGTCTRCGTGGACCGACAAG	5′-NC
DENV R1	CAGTTGACACRCGGTTTCTC	
DENV R2	GGGTTGATACGCGGTTTCTC	
DENV P	FAM-CGYCTWTCAATATGCTGAAACGCG-BHQ1	
CHIKV F	ACCATCGGTGTTCCATCTAAAG	nsP1
CHIKV R	GCCTGGGCTCATCGTTATT	
CHIKV P	HEX-ACAGTGGTTTCGTGTGAGGGCTAC-BHQ1	
ZIKV F	CCGCTGCCCAACACAAG	NS2A
ZIKV R	CCACTAACGTTCTTTTGCAGACAT	
ZIKV P	TAMRA-AGCCTACCTTGACAAGCAGTCAGACACTCAA-BHQ2	
YFV F	GCTAATTGAGGTGYATTGGTCTGC	5′-NC
YFV R	CTGCTAATCGCTCAAMGAACG	
YFV P	HEX-ATCGAGTTGCTAGGCAATAAACAC-BHQ	
OROV F	TCCGGAGGCAGCATATGTG	S segment
OROV R	ACAACACCAGCATTGAGCACTT	
OROV P	FAM-CATTTGAAGCTAGATACGG-MGB	
MAYV F	CACGGACMTTTTGCCTTCA	S segment
MAYV R	AGACTGCCACCTCTGCTKGAG	
MAYV P	HEX-ACAGATCAGACATGCAGG-MGB	

E: envelope gene. NS: non-structural protein. prM: precursor membrane gene. NC: non-coding region.
