# Supplementary material for: Integrated surveillance of arboviruses in febrile patients from the Brazilian Amazon reveals complex co-circulation dynamics and hidden viral diversity
Source: Rev Soc Bras Med Trop. 2026 Jul 17;59(Suppl 1):e0042-2026. doi: 10.1590/0037-8682-0042-2026 (PMC13379192; doi:10.1590/0037-8682-0042-2026)
Supplement: Supplementary material [file 1678-9849-rsbmt-59-s1-e0042-2026-md2.pdf]

Supplementary Table 2. Dengue virus serotype 1 primers used for the genome sequencing.

Primer	Sequence (5'-3')	Primer pool
DENV1_1_LEFT	AATATGCTGAAACGCGCGAGAA	1
DENV1_1_RIGHT	CCGTCTTCAAGAGTTCAATGTCCA	1
DENV1_2_LEFT	ACCCAGGATTCACGGTGATAGC	2
DENV1_2_RIGHT	ACCAGCAAATCTTGTCTGTTCCA	2
DENV1_3_LEFT	GGAAATACAGCTGACCGACTACG	1
DENV1_3_RIGHT	ACTGCAATGCACGTCATCGAAA	1
DENV1_4_LEFT	CAAGAAAGGAAGCAGCATAGGGA	2
DENV1_4_RIGHT	TTGATGGCAGCTGACATTAGCC	2
DENV1_5_LEFT	TGGAACATTTGGGAAGTTGAGGAC	1
DENV1_5_RIGHT	ACTTCTCTGGATGTTAGTCTGCG	1
DENV1_6_LEFT	TGGATGAACATTGTGGAAATCGAGG	2
DENV1_6_RIGHT	GCATGCCTCCAGCTATTAGTGG	2
DENV1_7_LEFT	AGTTGGCCCCTCAATGAAGGAA	1
DENV1_7_RIGHT	GCACTGACGTAGGTTCCACTTG	1
DENV1_8_LEFT	TCTCATATGGAGGAGGTTGGAGG	2
DENV1_8_RIGHT	AGCCTGAGTTCCATGATCTCTCA	2
DENV1_9_LEFT	ATAGCGGCCAGAGGGTACATCT	1
DENV1_9_RIGHT	TGTTCTCCTCCAACACCTGGTT	1
DENV1_10_LEFT	AAAGAGTGCAGCAATAGACGGG	2
DENV1_10_RIGHT	ATAGAGGGTCCAGGCTGAAGCT	2
DENV1_11_LEFT	TGTGGTGATAGGTTTGTTATTCATGATACT	1
DENV1_11_RIGHT	CTTTGGCTTCGGATCTGTCCAC	1
DENV1_12_LEFT	GGGAAACACTGGGAGAGAAATGG	2
DENV1_12_RIGHT	TGATCCTGATGGCTTGACCTCA	2
DENV1_13_LEFT	TGGAGCAAATGCAAAGAAAACATGG	1
DENV1_13_RIGHT	TGCACGACTTCCTTTTGCCTTT	1
DENV1_14_LEFT	ACTCAGCAAAAGAAGCAGTGGA	2
DENV1_14_RIGHT	GCATGGCACCACTATTTCCCTC	2
DENV1_15_LEFT	AGACGTGACCAGAGAGGAAGTG	1
DENV1_15_RIGHT	TCACTTGGTTTATGGCCACTTGT	1
