# Supplementary material for: Integrated surveillance of arboviruses in febrile patients from the Brazilian Amazon reveals complex co-circulation dynamics and hidden viral diversity
Source: Rev Soc Bras Med Trop. 2026 Jul 17;59(Suppl 1):e0042-2026. doi: 10.1590/0037-8682-0042-2026 (PMC13379192; doi:10.1590/0037-8682-0042-2026)
Supplement: Supplementary material [file 1678-9849-rsbmt-59-s1-e0042-2026-md3.pdf]

Supplementary Table 3. Dengue virus serotype 2 primers used for the genome sequencing.

Primer	Sequence (5'-3')	Primer pool
DENV2_1_LEFT	AGCAGATCTCTGATGAATAACCAACG	1
DENV2_1_RIGHT	TTTTTGCCATCGTCGTCACACA	1
DENV2_2_LEFT	TCGCTCCTTCAATGACAATGCG	2
DENV2_2_RIGHT	CCATTCTCAGCCTGCACTTGAG	2
DENV2_3_LEFT	ACATTGGTCACTTTCAAAAATCCCC	1
DENV2_3_RIGHT	TGAAGGGGATTCTGGTTGGAACT	1
DENV2_4_LEFT	ATAGTGGTTGCGTTGTGAGCTG	2
DENV2_4_RIGHT	CGGCAGCACCATTCTGTTATGA	2
DENV2_5_LEFT	TCATGCAGGCAGGAAAACGATC	1
DENV2_5_RIGHT	TCTCAAGAGTAGTCCAGCTGCA	1
DENV2_6_LEFT	TGGAAATCAGACCATTGAAAGAGAAAGA	2
DENV2_6_RIGHT	TGGTCAGTGTTTGTTCTTCCTCTT	2
DENV2_7_LEFT	CCAATCCTGTCAATAACAATATCAGAAGAT	1
DENV2_7_RIGHT	TGATGGCTGGGGTTTGGTATCT	1
DENV2_8_LEFT	AGATCGAAGATGACATTTTCCGAAAGA	2
DENV2_8_RIGHT	CCCATGTATATGTACTGGTCATTTTCATT	2
DENV2_9_LEFT	ATGCCAGTGACCCACTCTAGTG	1
DENV2_9_RIGHT	CCACCACTGTGAGGATGGCTAT	1
DENV2_10_LEFT	ACCAGAAAAACAGAGAACACCCC	2
DENV2_10_RIGHT	CCACTTCCTGGATTCCACTTTTCT	2
DENV2_11_LEFT	GGAGCTGGACTTCTCTTTTCCAT	1
DENV2_11_RIGHT	GACGTCCCAAGGTTTTGTCAGC	1
DENV2_12_LEFT	AGAGCATGAAACATCATGGCACT	2
DENV2_12_RIGHT	GTGCCTCTTGGTGTTGGTCTTT	2
DENV2_13_LEFT	TGGGACACAAGAATCACACTAGAAG	1
DENV2_13_RIGHT	CCGCACCATTGGTCTTCTCTTT	1
