# Supplementary material for: Integrated surveillance of arboviruses in febrile patients from the Brazilian Amazon reveals complex co-circulation dynamics and hidden viral diversity
Source: Rev Soc Bras Med Trop. 2026 Jul 17;59(Suppl 1):e0042-2026. doi: 10.1590/0037-8682-0042-2026 (PMC13379192; doi:10.1590/0037-8682-0042-2026)
Supplement: Supplementary material [file 1678-9849-rsbmt-59-s1-e0042-2026-md4.pdf]

Supplementary Table 4. Information regarding the dengue virus 1 (DENV-1) RT-qPCR positive and sequenced samples.

Sample ID	RT-qPCR Ct value	Genome coverage (CDS)*	Genome coverage (NT)*	Average depth	Identity (AA)*	Identity (NT)*	Genotype	Major lineage	Minor lineage	Sex	Age	Date	Location	GISAID ID
**CRN_050**	24.1	89.7%	85.1%	152.0	97.6%	83.7%	V	D	1.1	F	64	2021-04-27	Manaus, AM	EPI_ISL_20085827
**CRN_064**	19.9	94.2%	89.3%	161.2	98.2%	83.8%	V	D	1.1	F	31	2021-06-30	Manaus, AM	EPI_ISL_20085858
**CRN_087**	26.3	97.0%	91.9%	181.1	98.3%	83.9%	V	D	1.1	F	53	2022-03-09	Manaus, AM	EPI_ISL_20085828
**CRN_097**	22.2	95.4%	90.5%	76.6	98.3%	83.7%	V	D	1.1	F	24	2022-03-18	Manaus, AM	EPI_ISL_20085860
**CRN_099**	22.4	92.0%	87.3%	88.7	97.4%	83.5%	V	D	1.1	M	27	2022-03-22	Manaus, AM	EPI_ISL_20085968
**CRN_102**	35.1	NA	NA	NA	NA	NA	NA	NA	NA	M	23	2022-03-25	Manaus, AM	NA
**CRN_131**	28.5	96.5%	91.5%	172.4	98.3%	83.8%	V	D	1.1	M	45	2022-05-24	Manaus, AM	EPI_ISL_20085931
**CRN_144**	26.7	95.1%	90.2%	686.9	98.1%	83.8%	V	D	1.1	F	59	2022-06-07	Manaus, AM	EPI_ISL_20085859
**CRN_153**	19.4	99.2%	94.0%	1663.5	98.2%	83.8%	V	D	1	M	59	2022-06-09	Manaus, AM	EPI_ISL_20086603
**CRN_160**	19.6	99.6%	94.4%	1556.2	98.0%	83.8%	V	D	1.1	M	73	2022-06-23	Manaus, AM	EPI_ISL_20085932
**CRN_190**	22.9	99.6%	96.7%	2401.1	98.1%	83.8%	V	D	1.1	M	48	2022-08-25	Manaus, AM	EPI_ISL_20085998
**CRN_191**	22.2	96.6%	91.6%	310.8	98.3%	83.8%	V	D	1.1	F	21	2022-08-25	Manaus, AM	EPI_ISL_20086612
**CRN_410**	22.7	17.6%	16.8%	29.4	94.6%	82.0%	V	D	1.1	M	27	2022-11-22	Manaus, AM	EPI_ISL_20086608
**CRN_469**	36.9	NA	NA	NA	NA	NA	NA	NA	NA	F	29	2022-1205	Manaus, AM	NA
**CRN_514**	34.5	NA	NA	NA	NA	NA	NA	NA	NA	M	53	2022-12-15	Manaus, AM	NA

**CRN_588**	32.3	92.0%	87.2%	188.507	96.7%	83.3%	V	D	1.1	M	20	2023-01-09	Manaus, AM	EPI_ISL_20086606
**CRN_689**	29.6	94.5%	89.7%	543.31	97.7%	83.4%	V	D	1.1	M	35	2023-01-30	Manaus, AM	EPI_ISL_20086653
**CRN_701**	33.9	NA	NA	NA	NA	NA	NA	NA	NA	M	46	2023-02-03	Manaus, AM	NA
**CRN_122**	23.0	92.6%	89.2%	4.6	95.1%	90.5%	V	D	1.1	F	13	2022-06-23	Manaus, AM	
**CRN_159**	29.0	28.8%	28.3%	0.7	100%	99.9%	V	D	1.1	F	34	2022-06-23	Manaus, AM	

NT: nucleotide. CDS: coding DNA sequence. AA: amino acid. Ct: cycle quantification threshold. M: male. F: female. AM: Amazonas. *Coverage against the DENV-1 reference sequence NC_001477. NA: not applicable (not sequenced).
