# Supplementary material for: Integrated surveillance of arboviruses in febrile patients from the Brazilian Amazon reveals complex co-circulation dynamics and hidden viral diversity
Source: Rev Soc Bras Med Trop. 2026 Jul 17;59(Suppl 1):e0042-2026. doi: 10.1590/0037-8682-0042-2026 (PMC13379192; doi:10.1590/0037-8682-0042-2026)
Supplement: Supplementary material [file 1678-9849-rsbmt-59-s1-e0042-2026-md5.pdf]

Supplementary Table 5. Information regarding the dengue virus 2 (DENV-2) RT-qPCR positive and sequenced samples.

Sample ID	RT-qPCR Ct value	Genome coverage (CDS)*	Genome coverage (NT)*	Average depth	Identity (AA)*	Identity (NT)*	Genotype	Major lineage	Minor lineage	Sex	Age	Date	Location	GISAID ID
**CRN_042**	34.2	84.2%	80.0%	235.7	97.5%	85.0%	III	C	1.1	M	55	2021-04-19	Manaus, AM	EPI_ISL_20085785
**CRN_707**	35.4	77.9%	74.0%	176.1	97.9%	84.4%	II	F	1.1.2	M	39	2023-02-03	Manaus, AM	EPI_ISL_20085811
**CRN_695**	37.6	NA	NA	NA	NA	NA	NA	NA	NA	M	40	2023-01-31	Manaus, AM	NA

NT: nucleotide. CDS: coding DNA sequence. AA: amino acid. Ct: cycle quantification threshold. M: male. F: female. AM: Amazonas. *Coverage against the DENV-2 reference sequence NC_001474. NA: not applicable (not sequenced).
