# Supplementary material for: Integrated surveillance of arboviruses in febrile patients from the Brazilian Amazon reveals complex co-circulation dynamics and hidden viral diversity
Source: Rev Soc Bras Med Trop. 2026 Jul 17;59(Suppl 1):e0042-2026. doi: 10.1590/0037-8682-0042-2026 (PMC13379192; doi:10.1590/0037-8682-0042-2026)
Supplement: Supplementary material [file 1678-9849-rsbmt-59-s1-e0042-2026-md7.pdf]

Supplementary Table 7. DENV-2 genotype III complete genome sequences used for the phylogenetic analysis.

**GISAID ID**
EPI_ISL_18103434
EPI_ISL_18103529
EPI_ISL_18103429
EPI_ISL_18103500
EPI_ISL_18360929
EPI_ISL_808312
EPI_ISL_808316
EPI_ISL_808343
EPI_ISL_808335
EPI_ISL_808333
EPI_ISL_808337
EPI_ISL_808341
EPI_ISL_808323
EPI_ISL_808338
EPI_ISL_808331
EPI_ISL_18103508
EPI_ISL_18103492
EPI_ISL_18103468
EPI_ISL_14908638
EPI_ISL_18360823
EPI_ISL_14908643
EPI_ISL_14908654
EPI_ISL_14908671
EPI_ISL_14908672
EPI_ISL_18485750
EPI_ISL_18360822
EPI_ISL_18776249
EPI_ISL_18776245
EPI_ISL_773625
EPI_ISL_808306
EPI_ISL_808196
EPI_ISL_808307
EPI_ISL_939753
EPI_ISL_939764
EPI_ISL_808243
EPI_ISL_16215353
EPI_ISL_939760
EPI_ISL_939763
EPI_ISL_808589
EPI_ISL_808594
EPI_ISL_808177
EPI_ISL_14908658
EPI_ISL_16215355
EPI_ISL_18739846
EPI_ISL_18739867
EPI_ISL_18739852
EPI_ISL_18882331
EPI_ISL_18882329
EPI_ISL_17699816
EPI_ISL_939758
EPI_ISL_939757
EPI_ISL_18739849
EPI_ISL_939762
EPI_ISL_808591
EPI_ISL_14908642
EPI_ISL_14908653
EPI_ISL_18739856
EPI_ISL_808253
EPI_ISL_18240655
EPI_ISL_14908694
EPI_ISL_19507417
EPI_ISL_19507418
EPI_ISL_19507416
EPI_ISL_19507411

EPI_ISL_19507415
EPI_ISL_19507410
EPI_ISL_19507412
EPI_ISL_808382
EPI_ISL_14908641
EPI_ISL_19507400
EPI_ISL_19507359
EPI_ISL_808346
EPI_ISL_808322
EPI_ISL_808313
EPI_ISL_939750
EPI_ISL_939751
EPI_ISL_14908652
EPI_ISL_14908677
EPI_ISL_18739855
EPI_ISL_14908657
EPI_ISL_14908675
EPI_ISL_14908691
EPI_ISL_18103487
EPI_ISL_19507356
EPI_ISL_808256
EPI_ISL_808246
EPI_ISL_808174
EPI_ISL_939768
EPI_ISL_14908674
EPI_ISL_14908687
EPI_ISL_808260
EPI_ISL_808213
EPI_ISL_808262
EPI_ISL_18103489
EPI_ISL_16282284
EPI_ISL_16215354
EPI_ISL_18485679
EPI_ISL_17699777
EPI_ISL_939765
EPI_ISL_16212211
EPI_ISL_808168
EPI_ISL_808354
EPI_ISL_808205
EPI_ISL_808167
EPI_ISL_808261
EPI_ISL_808292
EPI_ISL_19507339
EPI_ISL_19507354
EPI_ISL_19507355
EPI_ISL_808613
EPI_ISL_19507360
EPI_ISL_19507361
EPI_ISL_19507353
EPI_ISL_17733676
EPI_ISL_17733678
EPI_ISL_16212210
EPI_ISL_808270
EPI_ISL_808226
EPI_ISL_18739851
EPI_ISL_775237
EPI_ISL_17699817
EPI_ISL_774869
EPI_ISL_774868
EPI_ISL_774867
EPI_ISL_808259
EPI_ISL_808269
EPI_ISL_808211
EPI_ISL_808263
EPI_ISL_808370
EPI_ISL_808373
EPI_ISL_14908670
EPI_ISL_14908651
EPI_ISL_19507362
EPI_ISL_14908666
EPI_ISL_14908647
EPI_ISL_14908656

EPI_ISL_939748
EPI_ISL_939756
EPI_ISL_14908692
EPI_ISL_14908673
EPI_ISL_14908649
EPI_ISL_14908650
EPI_ISL_808235
EPI_ISL_808366
EPI_ISL_808255
EPI_ISL_939759
EPI_ISL_939752
EPI_ISL_19507363
EPI_ISL_14908668
EPI_ISL_808219
EPI_ISL_18103464
EPI_ISL_939761
EPI_ISL_18861646
EPI_ISL_808176
EPI_ISL_808268
EPI_ISL_808350
EPI_ISL_808179
EPI_ISL_808597
EPI_ISL_14908640
EPI_ISL_808352
EPI_ISL_808251
EPI_ISL_16215356
EPI_ISL_808224
EPI_ISL_808595
EPI_ISL_808308
EPI_ISL_808368
EPI_ISL_939769
EPI_ISL_939771
EPI_ISL_939749
EPI_ISL_808230
EPI_ISL_18928530
EPI_ISL_17699772
EPI_ISL_19058589
EPI_ISL_18882332
EPI_ISL_14908644
EPI_ISL_808232
EPI_ISL_808181
EPI_ISL_772265
EPI_ISL_19049675
EPI_ISL_19049677
EPI_ISL_17599639
EPI_ISL_19070338
EPI_ISL_19070402
EPI_ISL_19070427
EPI_ISL_19070420
EPI_ISL_19070421
EPI_ISL_19070403
EPI_ISL_19070418
EPI_ISL_19070414
EPI_ISL_19070413
EPI_ISL_19070426
EPI_ISL_19070319
EPI_ISL_19070416
EPI_ISL_19070422
EPI_ISL_19070324
EPI_ISL_19070316
EPI_ISL_19070332
EPI_ISL_19070401
EPI_ISL_19070367
EPI_ISL_19070369
EPI_ISL_19070359
EPI_ISL_19070363
EPI_ISL_19070370
EPI_ISL_19070392
EPI_ISL_19070390
EPI_ISL_19070329
EPI_ISL_17498138
EPI_ISL_19026451

EPI_ISL_19070340
EPI_ISL_19070360
EPI_ISL_19070331
EPI_ISL_19070348
EPI_ISL_19070379
EPI_ISL_19070294
EPI_ISL_19070309
EPI_ISL_19070415
EPI_ISL_19070425
EPI_ISL_19070317
EPI_ISL_19070395
EPI_ISL_19070391
EPI_ISL_19026453
EPI_ISL_19070289
EPI_ISL_19070296
EPI_ISL_17600528
EPI_ISL_18403475
EPI_ISL_17600467
EPI_ISL_19070381
EPI_ISL_19070321
EPI_ISL_19070318
EPI_ISL_19070322
EPI_ISL_19070423
EPI_ISL_19070345
EPI_ISL_19070349
EPI_ISL_19070350
EPI_ISL_19070396
EPI_ISL_19136985
EPI_ISL_17600600
EPI_ISL_19136989
EPI_ISL_19136992
EPI_ISL_19136991
EPI_ISL_17600482
EPI_ISL_17600718
EPI_ISL_17600772
EPI_ISL_17498143
EPI_ISL_17600777
EPI_ISL_17498128
EPI_ISL_17498131
EPI_ISL_17600627
EPI_ISL_17600715
EPI_ISL_17600675
EPI_ISL_17600709
EPI_ISL_17600561
EPI_ISL_17600646
EPI_ISL_17600647
EPI_ISL_17600642
EPI_ISL_17498144
EPI_ISL_17498141
EPI_ISL_17600778
EPI_ISL_17600697
EPI_ISL_17600700
EPI_ISL_17600655
EPI_ISL_17498139
EPI_ISL_17600523
EPI_ISL_17600540
EPI_ISL_17600613
EPI_ISL_17600637
EPI_ISL_17600611
EPI_ISL_17600593
EPI_ISL_17600643
EPI_ISL_17600616
EPI_ISL_17600560
EPI_ISL_17600629
EPI_ISL_17600689
EPI_ISL_17600628
EPI_ISL_17600618
EPI_ISL_17600622
EPI_ISL_17600708
EPI_ISL_18861627
EPI_ISL_17600636
EPI_ISL_17600592

EPI_ISL_17600617
EPI_ISL_17600621
EPI_ISL_17600615
EPI_ISL_17600625
EPI_ISL_17600706
EPI_ISL_17600674
EPI_ISL_17600609
EPI_ISL_17600590
EPI_ISL_17600563
EPI_ISL_17600624
EPI_ISL_17498140
EPI_ISL_17600654
EPI_ISL_17600589
EPI_ISL_17600524
EPI_ISL_18861634
EPI_ISL_17600756
EPI_ISL_17600635
EPI_ISL_17600614
EPI_ISL_17600707
EPI_ISL_19507357
EPI_ISL_19507358
EPI_ISL_495007
EPI_ISL_715132
EPI_ISL_808198
EPI_ISL_808203
EPI_ISL_437480
EPI_ISL_10239375
EPI_ISL_438131
EPI_ISL_436798
EPI_ISL_11538425
EPI_ISL_438134
EPI_ISL_438129
EPI_ISL_438132
EPI_ISL_438130
EPI_ISL_443250
EPI_ISL_438136
EPI_ISL_438133
EPI_ISL_444992
EPI_ISL_443251
EPI_ISL_436797
EPI_ISL_438137
EPI_ISL_436799
EPI_ISL_444991
EPI_ISL_438135
EPI_ISL_443252
EPI_ISL_436794
EPI_ISL_939775
EPI_ISL_939774
EPI_ISL_773658
EPI_ISL_773652
EPI_ISL_773654
EPI_ISL_939784
EPI_ISL_773662
EPI_ISL_773661
EPI_ISL_773659
EPI_ISL_773664
EPI_ISL_773663
EPI_ISL_939781
EPI_ISL_939787
EPI_ISL_431883
EPI_ISL_431889
EPI_ISL_431892
EPI_ISL_431888
EPI_ISL_939792
EPI_ISL_773653
EPI_ISL_495011
EPI_ISL_496381
EPI_ISL_715397
EPI_ISL_496387
EPI_ISL_496388
EPI_ISL_496383
EPI_ISL_496379

EPI_ISL_431886
EPI_ISL_431890
EPI_ISL_431884
EPI_ISL_939776
EPI_ISL_431887
EPI_ISL_431893
EPI_ISL_773655
EPI_ISL_431885
EPI_ISL_773656
EPI_ISL_773660
EPI_ISL_773671
EPI_ISL_773670
EPI_ISL_773666
EPI_ISL_773667
EPI_ISL_773665
EPI_ISL_773669
EPI_ISL_939773
EPI_ISL_939780
EPI_ISL_773657
EPI_ISL_773672
EPI_ISL_773668
EPI_ISL_939777
EPI_ISL_496399
EPI_ISL_939790
EPI_ISL_939778
EPI_ISL_939788
EPI_ISL_939779
EPI_ISL_939791
EPI_ISL_939783
EPI_ISL_939794
EPI_ISL_17804515
EPI_ISL_17804519
EPI_ISL_17804524
EPI_ISL_17804508
EPI_ISL_17804529
EPI_ISL_17804528
EPI_ISL_17600666
EPI_ISL_17804523
EPI_ISL_17804506
EPI_ISL_17804513
EPI_ISL_17804514
EPI_ISL_17600663
EPI_ISL_17804534
EPI_ISL_17804532
EPI_ISL_17804518
EPI_ISL_17600690
EPI_ISL_19130003
EPI_ISL_19130009
EPI_ISL_17600657
EPI_ISL_17600669
EPI_ISL_17689852
EPI_ISL_17600660
EPI_ISL_19083105
EPI_ISL_17689849
EPI_ISL_17689876
EPI_ISL_17689848
EPI_ISL_19070388
EPI_ISL_808158
EPI_ISL_17689855
EPI_ISL_808155
EPI_ISL_3723495
EPI_ISL_3722706
EPI_ISL_808157
EPI_ISL_19082823
EPI_ISL_19070326
EPI_ISL_17689851
EPI_ISL_9437082
EPI_ISL_19070387
EPI_ISL_17600701
EPI_ISL_17498145
EPI_ISL_17600608
EPI_ISL_17600779

EPI_ISL_17600644
EPI_ISL_17600558
EPI_ISL_18081177
EPI_ISL_18081081
EPI_ISL_408706
EPI_ISL_18505877
EPI_ISL_18505881
EPI_ISL_19038102
EPI_ISL_19038090
EPI_ISL_18813678
EPI_ISL_18505893
EPI_ISL_19005061
EPI_ISL_18505890
EPI_ISL_17959704
EPI_ISL_18813676
EPI_ISL_18813682
EPI_ISL_17959710
EPI_ISL_18505884
EPI_ISL_19038108
EPI_ISL_17959708
EPI_ISL_17959723
EPI_ISL_19038061
EPI_ISL_19038063
EPI_ISL_18813679
EPI_ISL_18505873
EPI_ISL_18380640
EPI_ISL_18505891
EPI_ISL_716958
EPI_ISL_17600687
EPI_ISL_12665304
EPI_ISL_19032625
EPI_ISL_17783315
EPI_ISL_18720082
EPI_ISL_17600403
EPI_ISL_17783318
EPI_ISL_17783309
EPI_ISL_13154917
EPI_ISL_18081096
EPI_ISL_17783331
EPI_ISL_17783312
EPI_ISL_17783316
EPI_ISL_17783317
EPI_ISL_17783326
EPI_ISL_773579
EPI_ISL_17783320
EPI_ISL_436795
EPI_ISL_436796
EPI_ISL_773676
EPI_ISL_444993
EPI_ISL_18081123
EPI_ISL_773674
EPI_ISL_773675
EPI_ISL_18081148
EPI_ISL_18081166
EPI_ISL_18081089
EPI_ISL_18081102
EPI_ISL_18081155
EPI_ISL_18081080
