# Supplementary material for: Integrated surveillance of arboviruses in febrile patients from the Brazilian Amazon reveals complex co-circulation dynamics and hidden viral diversity
Source: Rev Soc Bras Med Trop. 2026 Jul 17;59(Suppl 1):e0042-2026. doi: 10.1590/0037-8682-0042-2026 (PMC13379192; doi:10.1590/0037-8682-0042-2026)
Supplement: Supplementary material [file 1678-9849-rsbmt-59-s1-e0042-2026-md8.pdf]

Supplementary Table 8. DENV-2 genotype II complete genome sequences used for the phylogenetic analysis.

GISAID ID
EPI-ISL-18870996
EPI-ISL-19179566
EPI-ISL-19358763
EPI-ISL-18871000
EPI-ISL-19358779
EPI-ISL-19358785
EPI-ISL-19115880
EPI-ISL-19115879
EPI-ISL-19358780
EPI-ISL-19376754
EPI-ISL-19376791
EPI-ISL-19376775
EPI-ISL-19376770
EPI-ISL-19376849
EPI-ISL-19471685
EPI-ISL-19150260
EPI-ISL-19541545
EPI-ISL-19541590
EPI-ISL-19058598
EPI-ISL-19058595
EPI-ISL-19076630
EPI-ISL-19333677
EPI-ISL-19358791
EPI-ISL-19358789
EPI-ISL-18485747
EPI-ISL-19058586
EPI-ISL-19058585
EPI-ISL-19058594
EPI-ISL-19552273
EPI-ISL-19552276
EPI-ISL-19254888
EPI-ISL-19609358
EPI-ISL-19179565
EPI-ISL-19358793
EPI-ISL-19376846
EPI-ISL-19541598
EPI-ISL-19058597
EPI-ISL-19241050
EPI-ISL-19376764
EPI-ISL-19115873
EPI-ISL-19333658
EPI-ISL-19179564
EPI-ISL-19058572
EPI-ISL-19507627
EPI-ISL-19507603
EPI-ISL-19609367
EPI-ISL-19664253
EPI-ISL-19664277
EPI-ISL-19664267
EPI-ISL-19219759
EPI-ISL-19115871
EPI-ISL-19115875
EPI-ISL-19115876
EPI-ISL-19115807
EPI-ISL-19115870
EPI-ISL-19115877
EPI-ISL-19358794
EPI-ISL-18870997
EPI-ISL-18871001
EPI-ISL-19210530
EPI-ISL-19058591
EPI-ISL-19058593
EPI-ISL-19552252
EPI-ISL-19552267

EPI-ISL-19609362
EPI-ISL-18679935
EPI-ISL-18679936
EPI-ISL-19609357
EPI-ISL-19358776
EPI-ISL-18840242
EPI-ISL-18840207
EPI-ISL-18840210
EPI-ISL-19609355
EPI-ISL-19597718
EPI-ISL-19167308
EPI-ISL-19413149
EPI-ISL-19167313
EPI-ISL-19413185
EPI-ISL-19664292
EPI-ISL-19664294
EPI-ISL-19664570
EPI-ISL-19439533
EPI-ISL-18945634
EPI-ISL-17733660
EPI-ISL-17733662
EPI-ISL-17733661
EPI-ISL-17733669
EPI-ISL-17733670
EPI-ISL-18714630
EPI-ISL-18714619
EPI-ISL-18714550
EPI-ISL-17734050
EPI-ISL-17734051
EPI-ISL-17734048
EPI-ISL-19210526
EPI-ISL-19167306
EPI-ISL-19167300
EPI-ISL-19597755
EPI-ISL-19240300
EPI-ISL-19471715
EPI-ISL-19240304
EPI-ISL-19471713
EPI-ISL-19240301
EPI-ISL-19471714
EPI-ISL-19240306
EPI-ISL-19471717
EPI-ISL-19240296
EPI-ISL-19471710
EPI-ISL-19234880
EPI-ISL-19234879
EPI-ISL-19597737
EPI-ISL-19210525
EPI-ISL-19597738
EPI-ISL-19597741
EPI-ISL-19240305
EPI-ISL-19471720
EPI-ISL-19240299
EPI-ISL-19471716
EPI-ISL-19240298
EPI-ISL-19471712
EPI-ISL-19240302
EPI-ISL-19471719
EPI-ISL-19234878
EPI-ISL-19234891
EPI-ISL-19240297
EPI-ISL-19471711
EPI-ISL-19234867
EPI-ISL-19234900
EPI-ISL-19597759
EPI-ISL-19115802
EPI-ISL-19115804
EPI-ISL-19609360
EPI-ISL-19234864
EPI-ISL-18714556
EPI-ISL-18714569
EPI-ISL-18714579

EPI-ISL-18714562
EPI-ISL-18714596
EPI-ISL-18714571
EPI-ISL-18714592
EPI-ISL-18714625
EPI-ISL-18714590
EPI-ISL-18714578
EPI-ISL-18714588
EPI-ISL-18714601
EPI-ISL-18714607
EPI-ISL-18714561
EPI-ISL-18714638
EPI-ISL-18714581
EPI-ISL-18714552
EPI-ISL-18714633
EPI-ISL-18714564
EPI-ISL-18714635
EPI-ISL-18714576
EPI-ISL-18714577
EPI-ISL-18714554
EPI-ISL-18714568
EPI-ISL-18714649
EPI-ISL-18714557
EPI-ISL-18714574
EPI-ISL-18714629
EPI-ISL-18714583
EPI-ISL-18714617
EPI-ISL-18714622
EPI-ISL-18714597
EPI-ISL-18714627
EPI-ISL-18714566
EPI-ISL-18714634
EPI-ISL-17734040
EPI-ISL-14908689
EPI-ISL-14908678
EPI-ISL-14908695
EPI-ISL-14908679
EPI-ISL-14908685
EPI-ISL-19358696
EPI-ISL-19597723
EPI-ISL-19358781
EPI-ISL-18953930
EPI-ISL-18953931
EPI-ISL-19333633
EPI-ISL-19358699
EPI-ISL-18679937
EPI-ISL-18679933
EPI-ISL-18679932
EPI-ISL-19609373
EPI-ISL-18871002
EPI-ISL-18679934
EPI-ISL-19629784
EPI-ISL-19629791
EPI-ISL-19629792
EPI-ISL-19358772
EPI-ISL-19358792
EPI-ISL-19150293
EPI-ISL-19115836
EPI-ISL-19358771
EPI-ISL-17983082
EPI-ISL-17983084
EPI-ISL-17673150
EPI-ISL-17673149
EPI-ISL-19541547
EPI-ISL-19541554
EPI-ISL-19541566
EPI-ISL-19295120
EPI-ISL-19295139
EPI-ISL-19609369
EPI-ISL-19541556
EPI-ISL-19552254
EPI-ISL-19552255

EPI-ISL-19552259
EPI-ISL-19552258
EPI-ISL-19333653
EPI-ISL-19219755
EPI-ISL-18882328
EPI-ISL-19219762
EPI-ISL-18485752
EPI-ISL-18882334
EPI-ISL-19552269
EPI-ISL-19597764
EPI-ISL-19219765
EPI-ISL-19219742
EPI-ISL-19552256
EPI-ISL-19552274
EPI-ISL-18219977
EPI-ISL-18219980
EPI-ISL-18219975
EPI-ISL-18219978
EPI-ISL-19471687
EPI-ISL-19240295
EPI-ISL-19471686
EPI-ISL-19240294
EPI-ISL-19234906
EPI-ISL-19234866
EPI-ISL-19234875
EPI-ISL-19234858
EPI-ISL-19471688
EPI-ISL-19240293
EPI-ISL-19234890
EPI-ISL-19234873
EPI-ISL-19234889
EPI-ISL-19325933
EPI-ISL-19325960
EPI-ISL-19325936
EPI-ISL-19325934
EPI-ISL-17699785
EPI-ISL-17983083
EPI-ISL-19358821
EPI-ISL-18714648
EPI-ISL-14908690
EPI-ISL-19219763
EPI-ISL-19541552
EPI-ISL-19541603
EPI-ISL-19358769
EPI-ISL-19609364
EPI-ISL-19459670
EPI-ISL-19459647
EPI-ISL-19459669
EPI-ISL-19459655
EPI-ISL-19459666
EPI-ISL-19459648
EPI-ISL-19597758
EPI-ISL-19597761
EPI-ISL-19597735
EPI-ISL-19115835
EPI-ISL-19115834
EPI-ISL-19115817
EPI-ISL-19115872
EPI-ISL-19115878
EPI-ISL-19358768
EPI-ISL-19471728
EPI-ISL-19474716
EPI-ISL-19210544
EPI-ISL-19210545
EPI-ISL-19210527
EPI-ISL-19376802
EPI-ISL-19241062
EPI-ISL-19376792
EPI-ISL-19376779
EPI-ISL-19376844
EPI-ISL-19597751
EPI-ISL-19082828

EPI-ISL-19376828
EPI-ISL-19333621
EPI-ISL-19179576
EPI-ISL-19376758
EPI-ISL-19376765
EPI-ISL-19333674
EPI-ISL-19597730
EPI-ISL-18403460
EPI-ISL-19083113
EPI-ISL-19167302
EPI-ISL-19167305
EPI-ISL-19167307
EPI-ISL-19210535
EPI-ISL-19210543
EPI-ISL-19597724
EPI-ISL-19459661
EPI-ISL-19664288
EPI-ISL-18953951
EPI-ISL-17733686
EPI-ISL-17733687
EPI-ISL-17733682
EPI-ISL-17733685
EPI-ISL-19325950
EPI-ISL-17983067
EPI-ISL-19115806
EPI-ISL-19115811
EPI-ISL-19115810
EPI-ISL-19115799
EPI-ISL-18714642
EPI-ISL-18474730
EPI-ISL-18474736
EPI-ISL-18474752
EPI-ISL-18714595
EPI-ISL-18474770
EPI-ISL-18714640
EPI-ISL-18776271
EPI-ISL-19179563
EPI-ISL-19358765
EPI-ISL-19358761
EPI-ISL-19609356
EPI-ISL-19629782
EPI-ISL-19629786
EPI-ISL-19358787
EPI-ISL-19358775
EPI-ISL-19239213
EPI-ISL-19471611
EPI-ISL-19609365
EPI-ISL-17699775
EPI-ISL-19541595
EPI-ISL-17699776
EPI-ISL-19220656
EPI-ISL-19210529
EPI-ISL-18776261
EPI-ISL-19597736
EPI-ISL-19210532
EPI-ISL-18776276
EPI-ISL-18485746
EPI-ISL-18485753
EPI-ISL-19376848
EPI-ISL-19376845
EPI-ISL-19376842
EPI-ISL-19664262
EPI-ISL-19459650
EPI-ISL-19597739
EPI-ISL-19333634
EPI-ISL-18743024
EPI-ISL-18510356
EPI-ISL-18776263
EPI-ISL-19376790
EPI-ISL-19376841
EPI-ISL-19376852
EPI-ISL-19376769

EPI-ISL-19376778
EPI-ISL-19376786
EPI-ISL-19376801
EPI-ISL-19321297
EPI-ISL-19597748
EPI-ISL-18776259
EPI-ISL-19506940
EPI-ISL-18776277
EPI-ISL-18776256
EPI-ISL-18776273
EPI-ISL-18485748
EPI-ISL-17983085
EPI-ISL-19381107
EPI-ISL-19442387
EPI-ISL-17699773
EPI-ISL-17699774
EPI-ISL-19376783
EPI-ISL-17983087
EPI-ISL-17983086
EPI-ISL-18776279
EPI-ISL-18776257
EPI-ISL-18776272
EPI-ISL-18776275
EPI-ISL-18714587
EPI-ISL-18714551
EPI-ISL-17734049
EPI-ISL-19376807
EPI-ISL-18742204
EPI-ISL-18510372
EPI-ISL-18510397
EPI-ISL-18689388
EPI-ISL-19204859
EPI-ISL-18505872
EPI-ISL-19038080
EPI-ISL-19038083
EPI-ISL-19161351
EPI-ISL-19161352
EPI-ISL-18813668
EPI-ISL-18813670
EPI-ISL-18813667
EPI-ISL-18474710
EPI-ISL-18474720
EPI-ISL-18474696
EPI-ISL-18474708
EPI-ISL-19257609
EPI-ISL-19161357
EPI-ISL-19161353
EPI-ISL-18403464
EPI-ISL-19474724
EPI-ISL-19474735
EPI-ISL-19474719
EPI-ISL-19474720
EPI-ISL-19376824
EPI-ISL-19376795
EPI-ISL-19376757
EPI-ISL-19376823
EPI-ISL-19376847
EPI-ISL-19376850
EPI-ISL-19058620
EPI-ISL-19370962
EPI-ISL-19058705
EPI-ISL-19541585
EPI-ISL-19541592
EPI-ISL-19507625
EPI-ISL-19507588
EPI-ISL-19507582
EPI-ISL-19507587
EPI-ISL-19507589
EPI-ISL-19664263
EPI-ISL-19376853
EPI-ISL-19541606
EPI-ISL-18909278

EPI-ISL-19541558
EPI-ISL-19609372
EPI-ISL-19333666
EPI-ISL-19541569
EPI-ISL-19058640
EPI-ISL-19058669
EPI-ISL-19370916
EPI-ISL-19131040
EPI-ISL-19085597
EPI-ISL-19085600
EPI-ISL-19370958
EPI-ISL-19370926
EPI-ISL-19058631
EPI-ISL-19552250
EPI-ISL-18909271
EPI-ISL-19541600
EPI-ISL-19376780
EPI-ISL-19376781
EPI-ISL-19376784
EPI-ISL-19376855
EPI-ISL-19541553
EPI-ISL-19376782
EPI-ISL-19358804
EPI-ISL-19358814
EPI-ISL-19358807
EPI-ISL-19358805
EPI-ISL-19358818
EPI-ISL-19358808
EPI-ISL-19358823
EPI-ISL-18909256
EPI-ISL-19115826
EPI-ISL-19115833
EPI-ISL-18909279
EPI-ISL-18909241
EPI-ISL-19370924
EPI-ISL-19664298
EPI-ISL-19358788
EPI-ISL-19541581
EPI-ISL-19597722
EPI-ISL-19541609
EPI-ISL-19370940
EPI-ISL-19370947
EPI-ISL-19370897
EPI-ISL-19370918
EPI-ISL-19370961
EPI-ISL-19058636
EPI-ISL-19058621
EPI-ISL-19358806
EPI-ISL-19058706
EPI-ISL-19058692
EPI-ISL-19370895
EPI-ISL-19541580
EPI-ISL-19358819
EPI-ISL-19541559
EPI-ISL-19370974
EPI-ISL-18909263
EPI-ISL-19058641
EPI-ISL-19459667
EPI-ISL-19541561
EPI-ISL-19358813
EPI-ISL-18909255
EPI-ISL-18909254
EPI-ISL-18909259
EPI-ISL-19058697
EPI-ISL-18909242
EPI-ISL-19058613
EPI-ISL-18909269
EPI-ISL-19058732
EPI-ISL-18909257
EPI-ISL-18909267
EPI-ISL-18909245
EPI-ISL-19664255

EPI-ISL-19664568
EPI-ISL-18909272
EPI-ISL-19370949
EPI-ISL-19370965
EPI-ISL-19115801
EPI-ISL-19115809
EPI-ISL-19115832
EPI-ISL-18909244
EPI-ISL-19664296
EPI-ISL-19435364
EPI-ISL-19370970
EPI-ISL-19609368
EPI-ISL-19358811
EPI-ISL-19058743
EPI-ISL-19058634
EPI-ISL-19058695
EPI-ISL-19370921
EPI-ISL-19058627
EPI-ISL-19058690
EPI-ISL-19058708
EPI-ISL-18909262
EPI-ISL-18909251
EPI-ISL-18909270
EPI-ISL-18909268
EPI-ISL-18909277
EPI-ISL-19058623
EPI-ISL-19541568
EPI-ISL-19370981
EPI-ISL-19541540
EPI-ISL-19058688
EPI-ISL-19370935
EPI-ISL-19597727
EPI-ISL-19370928
EPI-ISL-19058637
EPI-ISL-19234871
EPI-ISL-19234870
EPI-ISL-19234885
EPI-ISL-18953918
EPI-ISL-18909258
EPI-ISL-18909247
EPI-ISL-19370942
EPI-ISL-19026430
EPI-ISL-18984971
EPI-ISL-19266377
EPI-ISL-18714603
EPI-ISL-18714586
EPI-ISL-19358824
EPI-ISL-19358812
EPI-ISL-18909275
EPI-ISL-19358820
EPI-ISL-19471709
EPI-ISL-18485685
EPI-ISL-19358815
EPI-ISL-19552261
EPI-ISL-14908696
EPI-ISL-19597745
EPI-ISL-19597744
EPI-ISL-19358773
EPI-ISL-19376763
EPI-ISL-19629795
EPI-ISL-19609370
EPI-ISL-19333640
EPI-ISL-19541591
EPI-ISL-19541597
EPI-ISL-19358782
EPI-ISL-19115874
EPI-ISL-19115882
EPI-ISL-19358764
EPI-ISL-19358790
EPI-ISL-19541555
EPI-ISL-19541557
EPI-ISL-19295114

EPI-ISL-19239190
EPI-ISL-18714593
EPI-ISL-18714582
EPI-ISL-18714645
EPI-ISL-19541596
EPI-ISL-19295129
EPI-ISL-19179577
EPI-ISL-19664309
EPI-ISL-18485749
EPI-ISL-18485661
EPI-ISL-18714600
EPI-ISL-17699786
EPI-ISL-17699787
EPI-ISL-19471756
EPI-ISL-19507626
EPI-ISL-17983070
EPI-ISL-17983088
EPI-ISL-19541550
EPI-ISL-19541584
EPI-ISL-19541599
EPI-ISL-19541608
EPI-ISL-19541573
EPI-ISL-19541589
EPI-ISL-19342867
EPI-ISL-19342887
EPI-ISL-19680170
EPI-ISL-19220661
EPI-ISL-18485657
EPI-ISL-17983089
EPI-ISL-19179573
EPI-ISL-19179585
EPI-ISL-19664270
EPI-ISL-19664553
EPI-ISL-19609366
EPI-ISL-19507596
EPI-ISL-19295149
EPI-ISL-19541560
EPI-ISL-19541588
EPI-ISL-19541544
EPI-ISL-19541548
EPI-ISL-19541562
EPI-ISL-19058628
EPI-ISL-19058639
EPI-ISL-19680169
EPI-ISL-19220657
EPI-ISL-19664307
EPI-ISL-19220658
EPI-ISL-19179580
EPI-ISL-19179583
EPI-ISL-19664280
EPI-ISL-19179574
EPI-ISL-19664271
EPI-ISL-18714652
EPI-ISL-18485688
EPI-ISL-18714560
EPI-ISL-18713035
EPI-ISL-18714604
EPI-ISL-18714555
EPI-ISL-18714580
EPI-ISL-18714594
EPI-ISL-18714591
EPI-ISL-18714565
EPI-ISL-18714563
EPI-ISL-18713033
EPI-ISL-19325951
EPI-ISL-19325937
EPI-ISL-17734041
EPI-ISL-19325952
EPI-ISL-19325943
EPI-ISL-19325954
EPI-ISL-19115800
EPI-ISL-19376777

EPI-ISL-19376800
EPI-ISL-19376857
EPI-ISL-19376805
EPI-ISL-19376761
EPI-ISL-18776251
EPI-ISL-18776262
EPI-ISL-17733691
EPI-ISL-17733692
EPI-ISL-17733693
EPI-ISL-10239118
EPI-ISL-10238963
EPI-ISL-19161327
EPI-ISL-19161329
EPI-ISL-19038062
EPI-ISL-19038093
EPI-ISL-18813669
EPI-ISL-18505879
EPI-ISL-19474728
EPI-ISL-18689360
EPI-ISL-19474748
EPI-ISL-19474745
EPI-ISL-19474746
EPI-ISL-19474742
EPI-ISL-11539127
EPI-ISL-11538421
EPI-ISL-11539124
EPI-ISL-19673379
EPI-ISL-19682109
EPI-ISL-19682086
EPI-ISL-19682088
EPI-ISL-19333652
EPI-ISL-19333650
EPI-ISL-19682101
EPI-ISL-18474706
EPI-ISL-15997591
EPI-ISL-18474718
EPI-ISL-15997590
EPI-ISL-18377779
EPI-ISL-15997589
EPI-ISL-18377741
EPI-ISL-18377748
EPI-ISL-19234907
EPI-ISL-19471667
EPI-ISL-18474713
EPI-ISL-18377771
EPI-ISL-19032635
EPI-ISL-19673399
EPI-ISL-19521788
EPI-ISL-19521783
EPI-ISL-19211622
EPI-ISL-19211611
EPI-ISL-19210430
EPI-ISL-19673387
EPI-ISL-19673422
EPI-ISL-18474698
EPI-ISL-18474704
EPI-ISL-18474695
EPI-ISL-18474692
EPI-ISL-19474725
EPI-ISL-19474711
EPI-ISL-18474709
EPI-ISL-19474733
EPI-ISL-18917100
EPI-ISL-18363168
EPI-ISL-18917099
EPI-ISL-18363167
EPI-ISL-19474743
EPI-ISL-19474749
EPI-ISL-19474729
EPI-ISL-19333629
EPI-ISL-19474740
EPI-ISL-19474741

EPI-ISL-19474712
EPI-ISL-18813671
EPI-ISL-18505875
EPI-ISL-19063224
EPI-ISL-19063225
EPI-ISL-19063223
EPI-ISL-773039
EPI-ISL-18951544
EPI-ISL-18951492
EPI-ISL-19069855
EPI-ISL-19527335
EPI-ISL-19564040
EPI-ISL-18951526
EPI-ISL-18554123
EPI-ISL-18951522
EPI-ISL-19082826
EPI-ISL-19682123
EPI-ISL-19682077
EPI-ISL-19069848
EPI-ISL-18951502
EPI-ISL-18951503
EPI-ISL-18951541
EPI-ISL-18951551
EPI-ISL-18951487
EPI-ISL-18951558
EPI-ISL-18951529
EPI-ISL-18951493
EPI-ISL-18951530
EPI-ISL-18951528
EPI-ISL-18951545
EPI-ISL-18951571
EPI-ISL-18951482
EPI-ISL-18951572
EPI-ISL-19049680
EPI-ISL-18951486
EPI-ISL-18951483
EPI-ISL-18951494
EPI-ISL-19049671
EPI-ISL-18951511
EPI-ISL-18951542
EPI-ISL-18951510
EPI-ISL-18951565
EPI-ISL-18951505
EPI-ISL-18951520
EPI-ISL-18951556
EPI-ISL-18951537
EPI-ISL-18951485
EPI-ISL-18951516
EPI-ISL-18951566
EPI-ISL-18951543
EPI-ISL-18951496
EPI-ISL-18951484
EPI-ISL-18951479
EPI-ISL-19049674
EPI-ISL-18951538
EPI-ISL-19049682
EPI-ISL-18951575
EPI-ISL-18951557
EPI-ISL-19049679
EPI-ISL-19049673
EPI-ISL-18951553
EPI-ISL-18951531
EPI-ISL-18951561
EPI-ISL-18951519
EPI-ISL-18951527
EPI-ISL-18951535
EPI-ISL-19049669
EPI-ISL-19049670
EPI-ISL-18880859
EPI-ISL-18951524
EPI-ISL-19049681
EPI-ISL-18951488

EPI-ISL-18879914
EPI-ISL-18951564
EPI-ISL-18951514
EPI-ISL-18951569
EPI-ISL-18951504
EPI-ISL-18951562
EPI-ISL-18951489
EPI-ISL-18951491
EPI-ISL-18951490
