# Supplementary material for: Integrated surveillance of arboviruses in febrile patients from the Brazilian Amazon reveals complex co-circulation dynamics and hidden viral diversity
Source: Rev Soc Bras Med Trop. 2026 Jul 17;59(Suppl 1):e0042-2026. doi: 10.1590/0037-8682-0042-2026 (PMC13379192; doi:10.1590/0037-8682-0042-2026)
Supplement: Supplementary material [file 1678-9849-rsbmt-59-s1-e0042-2026-md9.pdf]

Supplementary Table 9. *Pegivirus hominis* sequences used for the phylogenetic analysis.

**GenBank ID**
LT009488.1
KC618399.1
OL672498.1
KM670100.1
U36380.1
KM670108.1
KM670109.1
KU685423.1
KP710604.1
AB008336.1
AB003291.1
LT009484.1
KC618400.1
AY949771.1
KC618398.1
AB018667.1
AB021287.1
AB003292.1
HQ331234.1
HQ331233.1
U94695.1
MH746815.1
AB003288.1
D90601.1
AB008342.1
D87708.1
MN215911.1
MH053116.1
MN215896.1
LT009486.1
JN127373.1
U63715.1
MK684252.1
MN551063.1
MH053115.1
MH053121.1
MN215895.1
MH053120.1
LT009483.1
KP259281.1
AX338086.1
MK291244.1

MN215908.1
U45966.1
LT009494.1
ON340918.1
LT009489.1
