# Supplementary material for: Integrated surveillance of arboviruses in febrile patients from the Brazilian Amazon reveals complex co-circulation dynamics and hidden viral diversity
Source: Rev Soc Bras Med Trop. 2026 Jul 17;59(Suppl 1):e0042-2026. doi: 10.1590/0037-8682-0042-2026 (PMC13379192; doi:10.1590/0037-8682-0042-2026)
Supplement: Supplementary material [file 1678-9849-rsbmt-59-s1-e0042-2026-md10.pdf]

Supplementary Table 10. *Erytroparvovirus primate* 1 sequences used for the phylogenetic analysis.

**GenBank ID**
DQ408304.1
MG572791.1
AY083234.1
DQ234778.2
MG572790.1
AY582124.2
DQ234779.2
MG572793.1
EF089225.1
KC013306.1
MG572794.1
EF089229.1
KP115297.1
OQ917657.1
OQ917658.1
OQ917659.1
EF089209.1
KC013325.1
MT534175.1
MT534176.1
Z68146.1
KP115323.1
MG572795.1
M13178.1
DQ293995.2
U38546.1
U38508.1
MG572789.1
MG572792.1
OK338446.1
OK338448.1
OK338449.1
OK338447.1
OK338459.1
AF162273.1
M24682.1
KP115318.1
AJ717293.1
AY064475.1
AY044266.2

DQ333426.1
AY582125.2
DQ234775.2
DQ234776.2
DQ234771.2
FJ265736.1
AJ249437.1
MH151117.1
DQ357064.1
DQ357065.1
DQ234769.2
