# Supplementary material for: Integrated surveillance of arboviruses in febrile patients from the Brazilian Amazon reveals complex co-circulation dynamics and hidden viral diversity
Source: Rev Soc Bras Med Trop. 2026 Jul 17;59(Suppl 1):e0042-2026. doi: 10.1590/0037-8682-0042-2026 (PMC13379192; doi:10.1590/0037-8682-0042-2026)
Supplement: Supplementary material [file 1678-9849-rsbmt-59-s1-e0042-2026-md12.pdf]

Supplementary Table 12. Information regarding *Erythroparvovirus primate* 1 identified by metagenomic shotgun sequencing.

Sample ID	Total reads*	Reads mapped to virus *	RPM*	Genome coverage (NT)*	Average depth	Identity (NT)*	Genotype	Sex	Age	Date	Location	GenBank_ID
CRN_113	1,964,592	66	33.6	33.8%	0.7	91.3%	III	F	11	2022-04-25	Manaus, AM	SAMN52632118
CRN_122	1,420,954	3,640	2562.5	90%	36.9	98.4%	III	F	13	2022-05-16	Manaus, AM	SAMN52632122
CRN_187	2,206,530	1,892,588	857743.9	100%	19,788.1	98.8%	III	F	60	2022-08-17	Manaus, AM	SAMN52632122

RPM: reads per million; NT: nucleotide; F: female; M: male; AM: Amazonas, Brazil.

*Total reads, reads mapped to virus, and RPM were obtained from CZID (Chan Zuckerberg ID platform).

Genome coverage, average depth, and identity values (NT) were calculated based on mapping to the *Erythroparvovirus primate* 1 reference genome (NC_000883.2). NA: not applicable (not sequenced or not detected).
